# Optimized Machine Learning for Autonomous Enzymatic Reaction Intensification in a Self‐Driving Lab

**DOI:** 10.1002/bit.70038

**Published:** 2025-08-04

**Authors:** Sebastian Putz, Niklas Teetz, Michael Abt, Pascal Jerono, Thomas Meurer, Matthias Franzreb

**Affiliations:** ^1^ Department for Bioengineering and Biosystems Karlsruhe Institute of Technology (KIT), Institute of Functional Interfaces (IFG) Eggenstein‐Leopoldshafen Germany; ^2^ Department for Electrobiotechnology Karlsruhe Institute of Technology (KIT), Institute of Process Engineering in Life Sciences (BLT) Karlsruhe Germany; ^3^ Department for Digital Process Engineering Karlsruhe Institute of Technology (KIT), Institute of Mechanical Process Engineering (MVM) Karlsruhe Germany

**Keywords:** automation, autonomous, digitalization, enzymes, machine learning, optimization algorithm, self‐driving labs

## Abstract

Optimizing enzymatic catalysis is crucial for enhancing the efficiency and scalability of many bioprocesses such as biotransformations, pharmaceutical synthesis, and food processing, as well as for improving the performance of analytical applications, including assays and biosensors. However, optimizing these reactions is challenging due to the multitude of interacting parameters such as pH, temperature, and cosubstrate concentration that require precise adjustment for maximum enzyme activity. Current optimization methods are often labor‐intensive and time‐consuming, especially when accounting for complex parameter interactions in highly dimensional parameter spaces. To overcome these challenges, we present a machine learning‐driven laboratory platform that enables rapid, data‐informed optimization of enzymatic reaction conditions in a fully automated environment. By conducting over 10,000 simulated optimization campaigns on a surrogate model generated via linear interpolation of experimentally obtained data, we identified and fine‐tuned the most efficient machine learning algorithm for optimizing enzymatic reactions. This allows the platform to autonomously determine optimal reaction conditions with minimal experimental effort and without human intervention. The effectiveness of our approach is demonstrated by the accelerated optimization of reaction conditions in a five‐dimensional design space across multiple enzyme‐substrate pairings. In conclusion, our self‐driving lab platform, equipped with a tailored optimization algorithm, offers a novel and superior alternative to traditional optimization methods. Moreover, the methodology for selecting the most efficient problem‐specific optimization algorithm can be extended to self‐driving lab platforms with broader applications.

## Graphical abstract

A machine learning‐powered self‐driving lab accelerates enzymatic reaction optimization by autonomously navigating complex parameter spaces. Through over 10,000 simulated campaigns, we identified an optimal algorithm for efficient, automated optimization. This approach rapidly fine‐tunes conditions across multiple reactions, offering a scalable, generalizable solution for diverse bioprocessing and analytical applications.

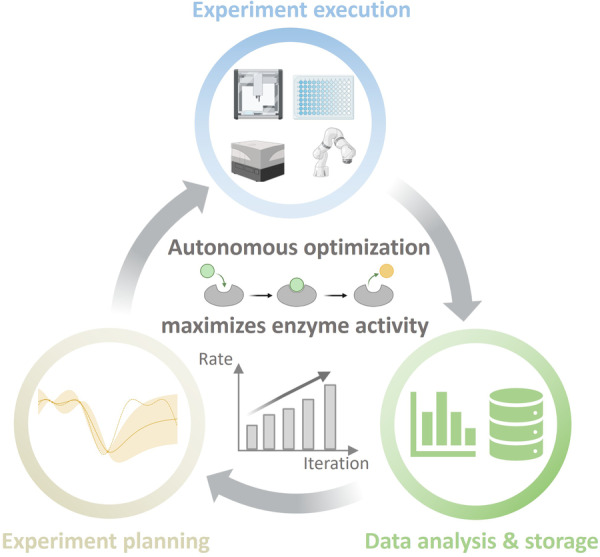

## 1 Introduction

Enzymatic reactions are fundamental to various industries, including fine chemical production (Hara et al. 2014; Liese and Villela Filho 1999; Thompson et al. 2019; Carrea and Riva 2000), pharmaceutical synthesis (Wu et al. 2021; Reetz et al. 2024; Kim et al. 2024; Simić et al. 2022; Chapman et al. 2018), food processing (Fernandes 2010; Yang et al. 2023; Wang et al. 2024), and analytical applications such as assays (Bisswanger 2014; Gan and Patel 2013; Asensio et al. 2008) and biosensors (Katz et al. 2001; Kilic et al. 2023; Melo et al. 2024) amongst others. These biocatalysts offer unique advantages over inorganic catalysts, such as high activities, reaction selectivity, substrate specificity, and stereo‐selectivity under mild reaction conditions (Rao et al. 2024). In addition, enzymatic processes are considered sustainable and environmentally friendly (Jegannathan and Nielsen 2013). For instance, in pharmaceutical synthesis, enzyme cascades facilitate the production of complex molecules with high selectivity, often surpassing traditional chemical methods in both efficiency and environmental compatibility (Siedentop and Rosenthal 2022).

However, optimizing enzymatic reactions is a complex endeavor due to the multitude of interacting parameters—such as pH, temperature, and cosubstrate concentration—that must be precisely adjusted to achieve maximum enzyme activity. This high‐dimensional design space, coupled with intricate parameter interactions, renders traditional optimization methods labor‐intensive and time‐consuming. For example, in enzyme cascade reactions, different optimal conditions for each enzyme involved can lead to challenges like side reactions and reduced overall efficiency (Siedentop et al. 2021; Onyeogaziri and Papaneophytou 2019).

To overcome these challenges in complex optimization problems, the concept of a self‐driving laboratory (SDL) has emerged. SDLs integrate automation with artificial intelligence (AI) to enhance experimental planning and execution (Canty et al. 2023; Abolhasani and Kumacheva 2023; Häse et al. 2019). SDLs can autonomously conduct experiments, analyze data, and iteratively refine conditions, thereby expediting the optimization process and reducing human intervention. This approach not only accelerates discovery but also improves reproducibility and efficiency in laboratory workflows (Bennett and Abolhasani 2022; Martin et al. 2023; Rooney et al. 2022; Soldatov et al. 2021). SDLs have already proven to be extremely effective tools for the accelerated discovery, optimization, and synthesis in the field of complex organic compounds (Steiner et al. 2019; Coley et al. 2019; Granda et al. 2018; Christensen et al. 2021; Ha et al. 2023), nanomaterials (Epps et al. 2020; Abdel‐Latif et al. 2021; Salley et al. 2020; Li et al. 2020a, 2020b; Volk et al. 2021), thin films (Harris et al. 2024; Nikolaev et al. 2014; MacLeod et al. 2022, 2020) as well as biomolecules and biosystems (King et al. 2004; Williams et al. 2015; Si et al. 2017; HamediRad et al. 2019; Kanda et al. 2022; Notin et al. 2024; Rapp et al. 2024), amongst others. Data‐driven optimization algorithms used in SDLs have already outperformed human scientists in certain applications such as chemical reaction optimization (Shields et al. 2021). However, to enable SDLs to effectively accelerate experimental optimization and discovery, selecting the appropriate optimization algorithm is essential (Hickman et al. 2023a, 2023b; MacLeod et al. 2023).

In this study, we present an autonomous, machine learning‐driven platform designed to rapidly optimize enzymatic reaction conditions within a fully integrated, self‐driving laboratory environment. Additionally, we introduce a method to identify efficient optimization algorithms to fine‐tune SDLs for specific tasks. This method includes an initial high‐throughput screening to generate an exemplary data set for the intended application of the SDL, which is then used for the in‐silico evaluation and optimization of multiple machine learning algorithms. For validation, the optimized machine learning algorithm is subsequently employed for optimization experiments on the SDL platform. After successful validation, the optimized algorithm can prospectively be used on the SDL to solve the intended task in the most efficient way. We exemplarily carried out these steps, for an SDL tailored to enzymatic reaction condition optimization. The results demonstrate that the optimized algorithm, Bayesian Optimization (BO) with a specific kernel and acquisition function, is highly generalizable across various enzyme‐substrate pairings. Using the fine‐tuned BO, enzymatic reaction conditions of multiple enzyme‐substrate pairings could be identified robustly and significantly accelerated compared to traditional methods.

## 2 Results and Discussion

### 2.1 Self‐Driving Lab Platform and Workflow Overview

**Figure 1:**
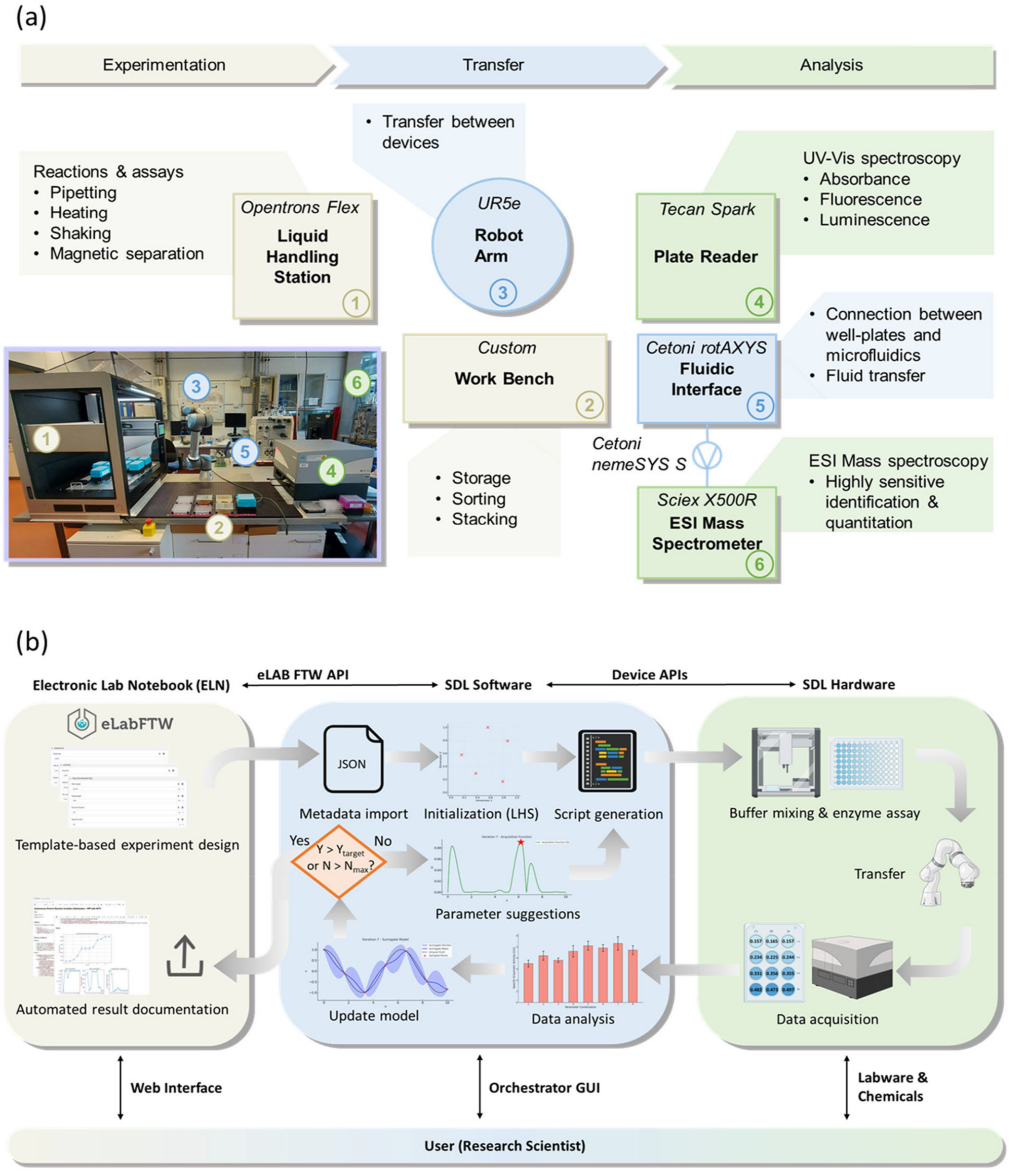
(a) Hardware schematic of the self‐driving lab platform for biochemical reaction optimization with photograph as inset. (b) Flow‐chart of the autonomous optimization workflow exemplified for the optimization of enzymatic reaction conditions.

We developed a self‐driving lab platform tailored for the autonomous optimization of biochemical reactions and analyses. Building upon our previous SDL work in solid‐phase extraction processes (Putz et al. 2024), this platform represents our first application to biocatalysis and bioanalytics, featuring substantial hardware and software advances. The first steps included setting up a hardware and software framework which allows for automated enzymatic reactions. The SDL currently integrates six laboratory devices and a custom‐built workbench, as depicted schematically and by a photograph in Figure 1a. At the core of the platform is a liquid handling station (OT Flex, Opentrons, USA), which provides pipetting, heating, shaking, gripper, and magnetic separation functionalities, enabling a wide range of (bio‐)chemical reactions and assays in well‐plate format. The custom‐designed workbench offers organized storage, sorting, and stacking for well‐plates, pipette tips, and liquid reservoirs. A 6‐DOF (six degrees of freedom) robotic arm (UR5e, Universal Robots, Denmark) equipped with an adaptive gripper (Hand‐E, Robotiq, Canada) and custom 3D‐printed fingers facilitates the automated transport and arrangement of labware and chemicals. Spectroscopic analysis is carried out using a multimode plate reader (Spark, Tecan, Switzerland), which supports UV‐vis spectroscopy, fluorescence, and luminescence measurements.

Additionally, the platform's well‐plate‐based system connects to a flow‐based microcapillary setup through a capillary positioning module (RotAXYS, Cetoni, Germany). Precision fluid transport is managed by high‐accuracy syringe pumps (nemeSYS S, Cetoni, Germany) and a flow‐selection valve module (6‐port Qmix V Valve, Cetoni, Germany), while integration with an electrospray‐ionization mass spectrometer (ESI‐MS) (X500‐R, Sciex, USA) coupled to an UPLC (Exion AD, Sciex, USA) enables highly sensitive detection and characterization of (bio‐)analytes. In this study, the platform primarily utilized the liquid handling station, robotic arm, and plate reader for automated colorimetric enzymatic assays. The modular architecture of the SDL, however, ensures flexibility and expandability, enabling the addition of specialized devices such as microreactors or alternative measurement modules to meet diverse experimental needs and further enhance its capabilities.

The integration of diverse commercial platforms into our Python‐based framework presented varying technical effort depending on vendor API support. Platforms with native Python APIs (e.g., Opentrons, Universal Robots) enabled direct integration, while proprietary systems utilizing C# interfaces (e.g., Tecan Spark SiLA Server, Sciex OS Control API) required the development of custom Python wrappers. These wrapper implementations typically required 2–3 days of development per device interface. The prevalence of Python APIs in laboratory instrumentation and the robust scientific computing ecosystem continue to support Python as the preferred framework for SDL backend development.

For a detailed explanation of the software architecture and APIs, we want to refer to our previous work (Putz et al. 2024) and the *Supporting Information* (Figure S2). In brief, it is a Python‐based modular framework, enabling high flexibility and easy integration of new devices and software modules. Key software advancements include the development of a Python wrapper for the Sciex OS API enabling automated analytical instrument control, and comprehensive end‐to‐end Electronic Laboratory Notebook (ELN) integration with eLabFTW using the eLabFTW Python API (API 2024). The ELN integration enables a seamless workflow from experimental design in the web‐based notebook through automated metadata import, experiment execution, and final upload of all data and auto‐generated reports back to the ELN for permanent documentation. Users can interact with the self‐driving lab using a custom‐programmed GUI (Supporting Information S1: Figure S1).

The workflow (Figure 1b) starts in the web interface of eLabFTW, where the user designs the experiment based on a predefined template. Each specific process, i.e., type of experiment, has its own template, where relevant metadata is entered into a structured input mask. Using the control software of the SDL, this metadata is imported to the local machine via the eLabFTW API. Following, the experiment is initialized: For autonomous iterative optimization processes an initial set of parameter combinations is generated using Latin Hypercube Sampling (LHS). For nonautonomous experiments, the parameter combinations are generated according to the experimental design specified in the metadata. Subsequently, the software generates the protocols to control the laboratory devices, namely the liquid handling station, the plate reader, and the ESI‐MS by embedding the relevant parameters and metadata into experiment‐specific templates. These templates are executed as part of a workflow script that orchestrates the operation of all connected laboratory devices during each experimental cycle.

For instance, in the optimization of enzymatic assays, the workflow starts with the preparation of buffers and assay mixtures by the liquid handler. Then the robot arm transfers the well‐plate into the plate reader for absorbance measurements. After the measurement, the robot arm either returns the plate back into the liquid handler or moves it to the storage bench if its fully used. The robot arm further prepares the setup for the next experimental cycle by replacing labware and reagents as needed. At the end of each experimental cycle, raw data is analyzed to perform calculations and generate plots. In the case of autonomous experiments, this data is used by the machine learning algorithm for the suggestion of the process parameter combinations for the next experimental cycle. The employed algorithms are either model‐based, such as BO, or model‐free and evolutionary like a Genetic Algorithm (GA). Using the parameter suggestions from the algorithm, new device control protocols are generated and the next experimental cycle begins.

In certain cases, human intervention is required after several cycles—for example, when labile compounds need special handling outside the SDL platform, or when the labware in the storage is depleted. Once the optimization process is complete, that is, the convergence criterion of the algorithm is met, a predefined target is reached (Y > Y_target_) or a specified number of experimental cycles is executed (N > N_max_), a final data evaluation is performed. This comprehensive, experiment‐type‐specific evaluation generates plots using data from all experimental cycles, highlighting improvements in the objective function and the influence of individual parameters. Finally, raw and processed data, including plots, are uploaded to the ELN using the API. The experimental documentation is completed by generating and uploading a written report, where placeholders in a predefined template are automatically replaced with the actual metadata and results.

### 2.2 Evaluating Machine Learning Algorithms for Efficient Enzymatic Reaction Optimization

Selecting an appropriate machine learning algorithm is critical for efficiently optimizing enzymatic reaction conditions. To ensure robust and rapid optimization, multiple machine learning approaches were systematically evaluated, identifying the most effective algorithm for guiding our self‐driving lab. To thoroughly assess different machine learning algorithms, first a comprehensive data set using high‐throughput screening was created, capturing a wide range of enzymatic reaction conditions for an exemplary reaction. Next, in‐silico experiments were conducted, simulating thousands of optimization campaigns to evaluate each algorithm's performance in navigating the complex parameter space.

#### 2.2.1 High‐Throughput Screening (HTPS) of Initial Enzymatic Reaction Rates

To provide a comprehensive data set for testing in‐silico different optimization algorithms in regard of their efficiency for process optimization of enzymatic reactions, a high‐throughput screening of a typical model reaction was conducted. The initial reaction rates of an unspecific peroxygenase AaeUPO‐PaDa‐I with a Twin‐Strep‐Tag (Twin‐Strep‐UPO) with the substrate 2,2‐Azino‐bis(3‐ethylbenzothiazoline‐6‐sulfonic acid) (ABTS) were screened under variation of the five key parameters pH, temperature, salt concentration, organic solvent concentration and cosubstrate concentration. Sodium sulfate (Na_2_SO_4_) was used as additional salt, acetonitrile (ACN) as additional organic solvent and hydrogen peroxide (H_2_O_2_) as cosubstrate.

**Figure 2:**
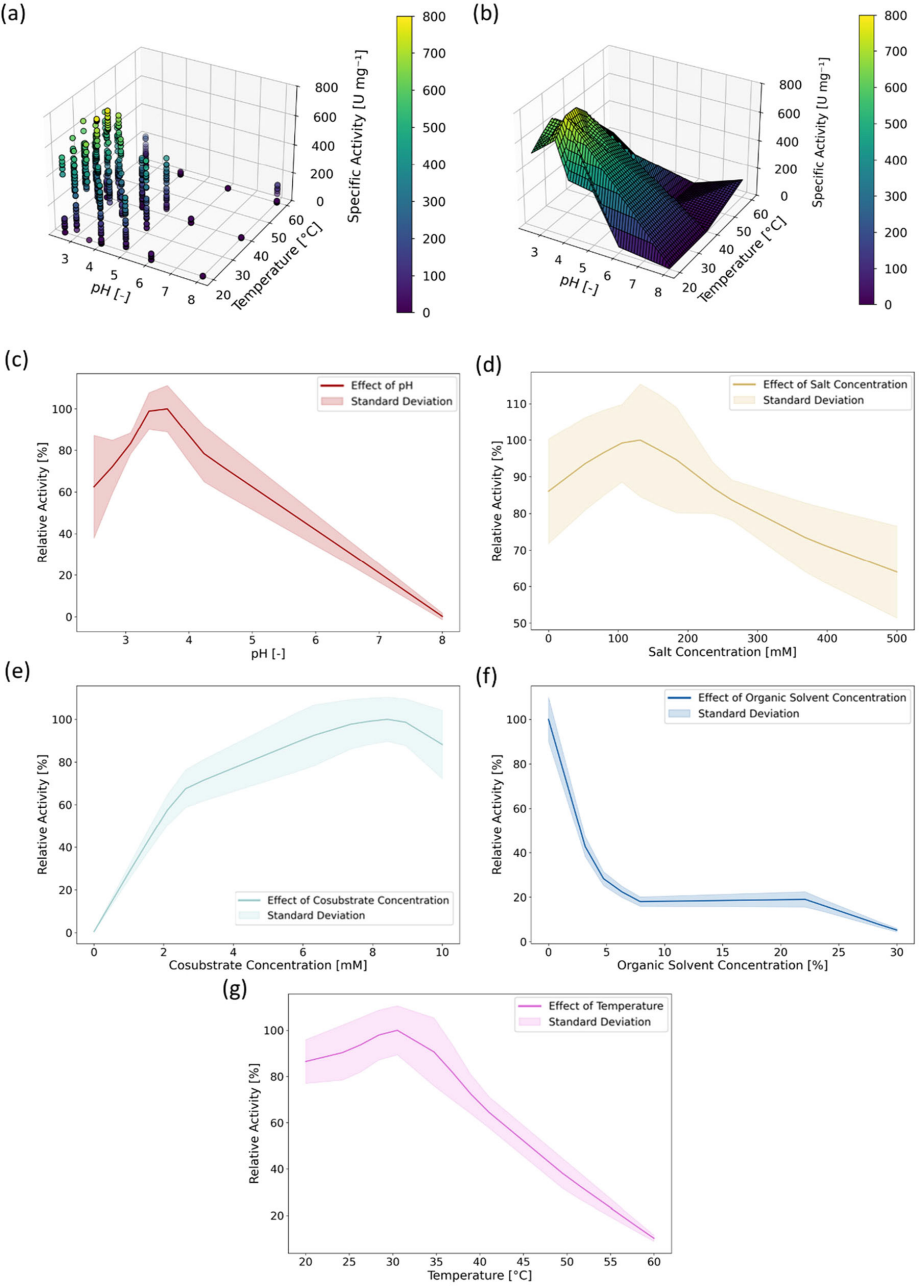
(a) Specific enzymatic activities of the AaeUPO PaDa‐I with Twin‐Strep‐Tag as a function of pH and temperature from the high throughput screening under variation of pH, temperature, cosubstrate (H_2_O_2_), salt (Na_2_SO_4_) and organic solvent (ACN) concentration. The values are the mean of replicated experiments (n* =* 2). (b) Response surface of the enzymatic activity as a function of pH and temperature obtained through linear interpolation of mean experimental data points. (c–g) One dimensional projection of the interpolated response surface for varying pH (c), salt concentration (d), cosubstrate concentration (e), organic solvent concentration (f) and temperature (g) while keeping all other parameters fixed at the optimal values. The fixed values are pH = 3.5, T* =* 30°C, with cosubstrate concentration cH_2_O_2_ *=* 8.75 mM, salt concentration cNa_2_SO_4_ *=* 120 mM, and organic solvent concentration c_ACN_ *=* 0% (v/v).

In Figure 2a, the mean values of the initial reaction rates are shown over pH and temperature for all investigated parameter combinations. The initial reaction rate presents a maximum at pH = 3.5, T = 30°C, with cosubstrate concentration cH_2_O_2_ = 8.75 mM, salt concentration cNa_2_SO_4_ = 120 mM, and organic solvent concentration c_ACN_ = 0% (v/v). The peak activity at the maximum is 790.4 ± 80.6 U mg^‐1^. Due to the iterative Design of Experiments (DoE)‐based sampling, which employed three sequential 3‐Level‐Full‐Factorial designs, the regions closer to the optimal conditions are sampled more densely than those farther away.

To ensure all possible parameter combinations within the design space were computationally accessible from the finite set of recorded data points, linear interpolation was applied for the in‐silico experiments (see Section 2.3). The resulting interpolated response surface as a function of pH and temperature is illustrated in Figure 2b. A critical discussion of the interpolation model's confidence, associated uncertainty, and a demonstration of its suitability for algorithm benchmarking is provided in the *Supporting Information, Section 4. Linear interpolation surrogate model confidence and uncertainty quantification)*.

To assess the influence of individual parameters, one dimensional projections of the interpolated response surface were generated (Figure 2c–g). These show the influence of the individual parameters while keeping the other four parameters constant at their optimal values. The initial enzymatic reaction rate is highest at pH = 3.5 and decreases to 61.1% at pH = 2.4% and 2.0% at pH = 8, the extremes of the design space (Figure 2c). The pH dependency of enzymatic activity typically exhibits a bell‐shaped curve, with a steeper decline in activity as the pH increases beyond its optimal value. The approximately linear relationship between pH = 4 and pH = 8 observed in Figure 2c is a result of interpolation in sparsely populated regions of the 5‐dimensional design space. Similarly, the temperature exhibits a single optimum at T = 30°C and the relative activity declines to 85.5% at T = 20°C and 10.2% at T = 60°C (Figure 2g). Increasing the concentration of the cosubstrate H_2_O_2_ from 0 to 8.5 mM continually increases the reaction rate (Figure 2e) from 0.3% to 100%. Increasing H_2_O_2_ concentration further to 10 mM reduces the reaction rate to 88.1% of the peak value. At 0 mM H_2_O_2_, the reaction rate approaches zero, as no cosubstrate for the reaction is available. As shown in Figure 2f, the reaction rate is maximal at 0% (v/v) ACN and decreases to 4.8% when increasing the organic solvent concentration to 30% (v/v). On average, adding additional 125 mM Na_2_SO_4_, yields the highest activity (Figure 2d). The relative activity is slightly decreased to 87.2% when no Na_2_SO_4_ is added to the buffer and more strongly decreased to 64.8% when a high concentration of 500 mM Na_2_SO_4_ is added.

The identified optimal reaction conditions for the substrate ABTS align well with previous studies on recombinant AaeUPO enzymes, which typically report pH optima between 3.0 and 4.0 and temperature optima around 30°C (Molina‐Espeja et al. 2014, 2015; Yan et al. 2024). In this study, the enzyme was not pre‐incubated at the target temperature; instead, the initial activity was measured immediately after the enzyme was added to the reaction mixture. Interestingly, higher temperatures exceeding 30°C still led to reduced activity, likely due to the rapid thermal deactivation kinetics of the enzyme. UPOs typically show a decreasing activity in ABTS assay with increasing organic solvent concentration (Martin‐Diaz et al. 2021), which could also be observed in the conducted screening. An H_2_O_2_ concentration of 8.75 mM was found to be optimal to maximize the initial enzymatic reaction rate. This concentration is substantially higher than 2 mM which is typically used for ABTS assays with UPOs (Dolz et al. 2023; Bormann et al. 2015). While elevated H_2_O_2_ levels enhance the reaction rate, they can simultaneously promote enzyme inactivation (Hofrichter et al. 2015). As this study focused solely on the initial reaction rate within a 60‐second timeframe, 8.75 mM appears to strike the most favorable balance between enzyme inactivation and reaction kinetics. Additionally, the screening suggests adding 120 mM Na_2_SO to the phosphate‐citrate buffer maximizes the initial enzymatic reaction rate. It can be hypothesized that additional salt in the reaction buffer could favorably alter the electrostatic enzyme‐enzyme and enzyme‐substrate interactions to increase the enzymatic reaction rate. The average coefficient of variation (CV) for the ABTS assay was 13.2%, which is comparable to reported reference values of approximately 12% (Dolz et al. 2023).

#### 2.2.2 In‐Silico Testing and Fine‐Tuning of Optimization Algorithms

To identify the most effective algorithm for this optimization problem—maximizing the initial enzymatic reaction rate by varying reaction conditions—a screening and fine‐tuning of several optimization algorithms was conducted. These algorithms were evaluated in simulated experimental optimization campaigns (see *4. Experimental Section*), where both the number of iterations and the highest enzymatic activity achieved were tracked. Detailed descriptions of the utilized optimization algorithms, flow charts and explanations of the investigated hyperparameters are presented in the *Supporting Information*.

**Figure 3:**
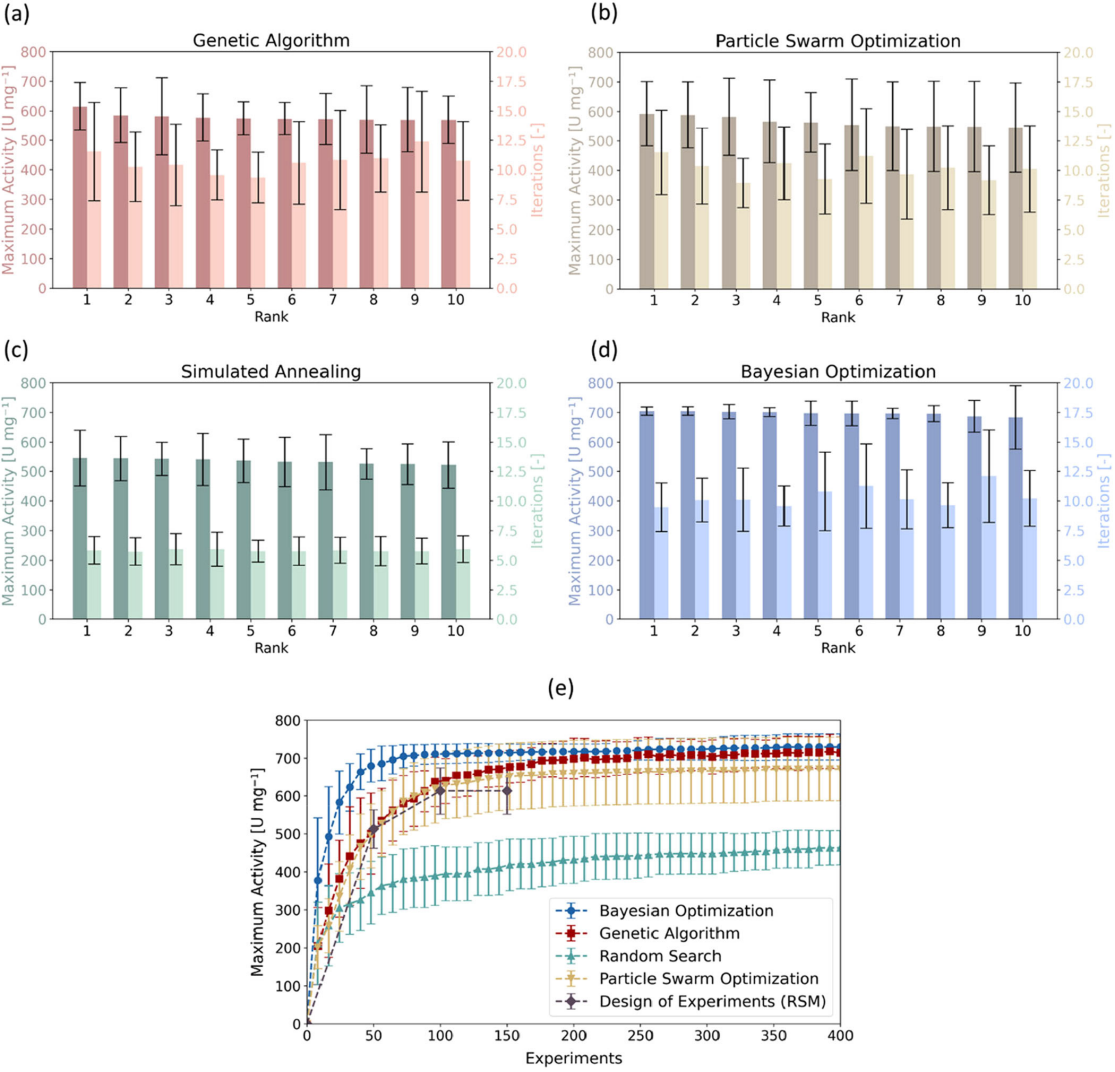
Comparison of optimization algorithms by simulated optimization campaigns. (a–d): Maximal enzymatic activity and number of iterations for the top 10 best performing algorithms for (a) Genetic Algorithm (GA), (b) Particle Swarm Optimization (PSO), (c) Simulated Annealing (SA) and (d) Bayesian Optimization (BO). All values represent the mean and standard deviation averaged over 30 optimization campaigns using the same 30 initial parameter combinations for all algorithms. (e) Comparison of the maximal activity for the best‐performing BO, GA and PSO to Random Search (RS) and Response Surface Modeling (RSM) over 400 iterations. All values represent the mean and standard deviation averaged over 30 optimization campaigns using the same 30 initial parameter combinations for all algorithms. For RSM, the experimental design was fixed but the optimization was run 30 times, deviations arise from the noise in the simulated experiments.

Figure 3a–d display the highest scores and corresponding iteration counts for the top 10 variants of the Genetic Algorithm (GA), Particle Swarm Optimization (PSO), Simulated Annealing (SA), and Bayesian Optimization (BO). Among these, SA exhibited the lowest performance, achieving a maximum activity of 527.4 U mg⁻¹ within 5.83 iterations averaged over the top 10. PSO performed better, with a mean maximum activity of 563.8 U mg⁻¹ over an average of 10.11 iterations. GA ranked second, reaching an average best score of 578.6 U mg⁻¹ across 10.66 iterations. BO was the most effective algorithm, achieving a superior average maximum activity of 696.8 U mg⁻¹ in 10.33 iterations.

To gain insights into the hyperparameter tuning, in Tables 1, 2, 3, 4, the hyperparameters of the top 5 performing algorithm variants are shown for the GA, PSO, BO and SA. For the GA, an efficient setup included migrating 1 to 2 top individuals into subsequent generations (referred to as elitism), a moderate mutation extent (0.3–0.6), random crossover during recombination, and rank‐based selection. The mutation probability seems to have a weak effect, as all investigated values are present in the top 5 (Table 1). These settings balance exploration and exploitation, maintaining diversity while ensuring convergence. To construct an efficient PSO algorithm for the specific optimization problem, a high initial inertia weight w_0_ (0.9−1.1), a slow decay factor for the inertia c_d_ (0.85–0.95) and larger cognitive than social factor, c_1_ ≥ c_2_, should be chosen (Table 2). This configuration emphasizes individual particle exploration, which seems beneficial in the investigated search space. BO performed best with a Matérn kernel and an acquisition function that is either EI, UCB or PI (Table 3). These choices facilitate a balance between exploring new areas and exploiting known promising regions. Briefly, the acquisition functions control how the algorithm navigates the trade‐off between sampling regions with high predicted activity (exploitation) and exploring uncertain regions (exploration). The choice of kernel, such as Matérn, influences the flexibility and smoothness of the surrogate model, allowing it to capture varying levels of complexity in the underlying activity landscape (for detailed explanations, see *Supporting Information*, *Section 2. Utilized optimization algorithms*). For SA no clear trend could be observed, except that a large initial step size of 0.8 seems most effective for the optimization task (Table 4).

**Table 1:** Hyperparameters of the top five best performing genetic algorithm (GA) variants from the in‐silico optimization algorithm testing.

Rank	Elitsm	Mutation probability	Mutation extent	Cross‐over	Selection
1	2	0.4	0.3	Random	rank
2	1	0.1	0.3	Random	rank
3	1	0.2	0.3	Random	rank
4	1	0.1	0.6	Random	rank
5	1	0.8	0.3	single point	rank

**Table 2:** Hyperparameters of the top 5 best performing particle swarm optimization (PSO) variants from the in‐silico optimization algorithm testing.

Rank	w_0_	c_d_	c_1_	c_2_
1	0.9	0.95	2.5	1.0
2	0.9	0.95	1.5	1.0
3	1.1	0.95	1.5	1.5
4	0.9	0.95	2.0	1.0
5	1.1	0.85	2.5	1.5

**Table 3:** Hyperparameters of the top 5 best performing Bayesian optimization (BO) variants from the in‐silico optimization algorithm testing.

Rank	Kernel Function	Acquisition function
1	Matérn 32	EI
2	Matérn 32	UCB
3	Matérn 12	EI
4	Matérn 12	UCB
5	Matérn 32	PI

**Table 4:** Hyperparameters of the top five best performing simulated annealing (SA) variants from the in‐silico optimization algorithm testing.

Rank	T_0_	c_T_	S_0_
1	380	0.7	0.8
2	150	0.6	0.8
3	150	0.7	0.8
4	380	0.9	0.8
5	75	0.5	0.8

In Figure 3e the performances of the top GA, PSO, and BO algorithms are compared with Random Search (RS) and Response Surface Modeling (RSM) as benchmarks. Over 400 experiments (50 iterations), BO achieved the fastest convergence, reaching an average maximum activity of 695.9 ± 37.1 U mg⁻¹ within 64 experiments (8 iterations). It plateaued at 729.7 ± 34.43 U mg⁻¹ after 400 experiments. The best parameter combinations identified across all optimization runs are clustered closely around the single optimum in the parameter space with values of pH = 3.5 ± 0.0, T = 20.9 ± 2.5°C, cH_2_O_2_ = 9.6 ± 0.8 mM, cNa_2_SO_4_ = 211.7 ± 132.3 mM, and c_ACN_ = 0 ± 0.0% (v/v). In contrast, GA and PSO converged more slowly. After 64 experiments, their maximal activities were similar (560.9 ± 80.0 U mg⁻¹ for GA and 558.1 ± 88.2 U mg⁻¹ for PSO), while both continued to improve gradually. GA eventually plateaued at 714.0 ± 42.4, while PSO stabilized at 671.3 ± 84.3. Thus, the GA and PSO can find optima in a similar range than the BO, but converge at a slower rate. Therefore, the lack‐of‐improvement convergence criterion (less than 5% improvement over 5 rounds) terminated the GA and PSO in the grid‐search algorithm testing before reaching the maximum score. Additionally, the BO has the lowest standard deviation averaged over the 30 initial generations indicating only little dependence of maximal activity on the initial conditions. In contrast, PSO has the largest standard deviation, indicating a stronger dependence on the initial parameter combinations.

In comparison, a 3‐step Response Surface Modeling (RSM) identifies a maximal score of 615.9 ± 54.7 in 150 experiments. Notably, the quadratic interaction models fitted at each round of the RSM procedure achieved coefficients of determination (R²) of 0.762 ±  0.005 in the first round, 0.826  ±  0.007 in the second round, and 0.860  ±  0.013 in the final round. This progressive improvement in R² demonstrates that as RSM iteratively narrows the search region, the local fit to the experimental data improves. However, the overall lower maximal activity achieved by RSM compared to model‐free (GA, PSO, SA) or GP‐based (BO) optimization algorithms highlights a key limitation: the quadratic model, despite good fits in the sampled region, does not fully capture the complex parameter dependencies present in the enzymatic system and can thus fail to efficiently guide the search toward the global optimum. Up to 100 experiments, the RSM performs comparably to the GA and PSO, which then outperform the RSM by converging to a higher maximal score at a slow rate. The BO plateaus at a similar number of experiments as the RSM, approximately 100, however reaching a higher maximal enzymatic activity. This can be explained by the rigid quadratic interaction model used in the RSM, which seems not to accurately cover the underlying relationship of the parameters on the enzymatic reaction rate. Unlike RSM, GA and PSO are model‐free optimization methods that do not rely on fitting a predefined equation or model to the data. This allows these algorithms to explore the parameter space more flexibly. On the other hand, BO fits a model—specifically, a Gaussian process (GP)—that leverages all available data to predict the objective function. The GP model is highly flexible, capable of adapting to complex, nonlinear relationships in the parameter space. The comparison to RS shows that either method is more effective than random sampling within the design space. This demonstrates the importance of leveraging information‐driven optimization techniques for effectively exploring and exploiting the design space.

Overall, these results indicate among all tested algorithms BO is most effective for optimizing enzymatic reaction conditions on the provided data set. This can be attributed to the inherent strength of the GP model, which provides a probabilistic framework for modeling the objective function. By quantifying uncertainty and incorporating prior knowledge, BO efficiently balances exploration and exploitation. The Gaussian process enables BO to focus experimental efforts on the most promising regions of the parameter space, adapting dynamically as new data becomes available. This approach is particularly effective for capturing complex relationships in the enzymatic reaction rate, leading to faster convergence to an optimal solution compared to other algorithms. BO is among the most common experimental planning strategies for SDLs (Tom et al. 2024). It has proven to be a very effective optimization algorithm in SDLs for diverse applications including protein engineering (Rapp et al. 2024), adhesives (Rooney et al. 2022), nanoparticles (Vaddi et al. 2022), metal‐organic frameworks (Xie et al. 2021), thin films (Ohkubo et al. 2021) and battery electrolytes (Dave et al. 2020).

Given the similarity of optimization challenges across enzyme‐substrate systems, we hypothesize that the fine‐tuned BO algorithm can be broadly applied to optimize reaction conditions for various enzymes and substrates. To test this hypothesis, we conducted autonomous optimization campaigns on the SDL with different enzyme‐substrate pairings (see *Section 2.4*).

### 2.3 Autonomous Enzymatic Reaction Optimization With Fine‐Tuned Algorithm

The best‐performing algorithm from the in‐silico testing, Bayesian Optimization (BO) with a Matérn 32 kernel and Expected Improvement (EI) acquisition function, was deployed on the SDL to conduct real experiments for autonomously optimizing the reaction conditions of enzymes with different substrates. Each experimental campaign was initialized de novo without prior knowledge, using LHS to generate the initial parameter sets for each new enzyme–substrate combination. After each experimental cycle, the algorithm suggested eight new parameter combinations to test in the subsequent cycle.

**Figure 4:**
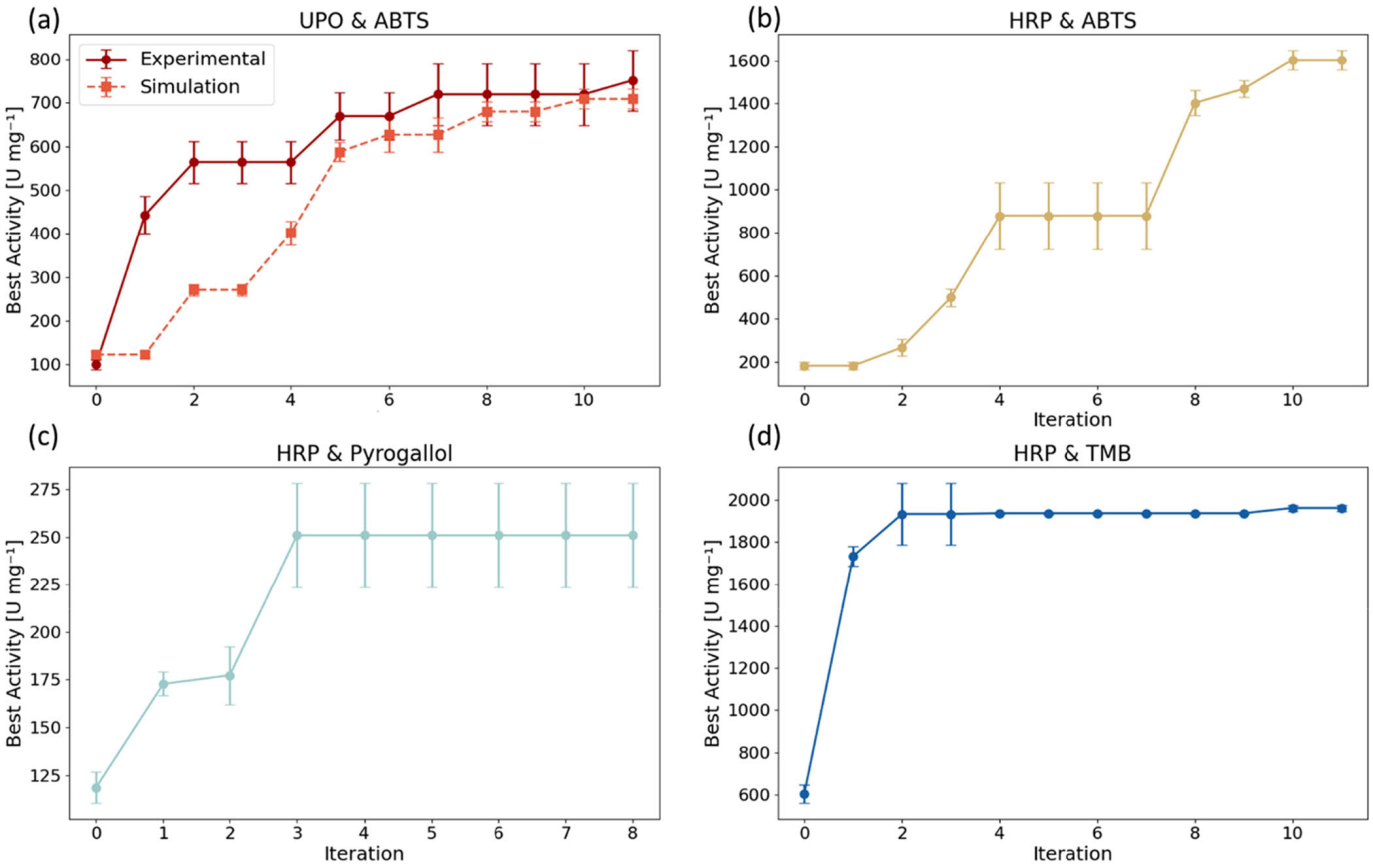
Autonomous optimization of enzymatic reaction conditions in real experiments on the SDL platform. (a): Overall highest enzymatic activity versus iterations for UPO ‐ ABTS and comparison to simulated experiment. The same initial parameter combinations were used for experiment and simulation in iteration 0. (b–d): Overall highest enzymatic activity versus iterations for HRP ‐ ABTS (b), HRP–pyrogallol (c) and HRP‐ TMB (d). All values represent the mean and standard deviation of replicated experiments (*n* = 3). In each iteration 8 different parameter combinations were tested.

To validate the results and compare simulated and experimental outcomes, the reaction conditions for the Twin‐Strep‐UPO with ABTS as the substrate were optimized first. Figure 4a displays the highest enzymatic reaction rate over the iterations for this UPO‐ABTS pairing. In the experiment, the highest enzymatic activity increased from 133.3 ± 11.7 U mg^‐1^ in the initial cycle to 708.2 ± 69.7 U mg^‐1^ by iteration 11. For the simulated experiment with the same initial parameter combinations, the enzymatic activity increased from 123.0 ± 7.8 to 708.6 ± 22.5 U mg^‐1^ during the optimization. Although in the experiments with the SDL the overall highest activity initially improved faster, experiment and simulation align closely from iteration 5 onwards. Comparing the optimal conditions identified by the SDL and the simulated experiments (Table 5), small differences can be found. While both identify an optimal pH of 3.4 and organic solvent concentration of 0% (v/v), the SDLs optimum has a lower temperature (20°C vs. 30°C in the simulation), a lower salt concentration (0 mM vs. 125 mM) and a higher H_2_O_2_ concentration (10.0 mM vs. 8.0 mM). These differences can be explained by experimental noise and inaccuracies of the simulated activities obtained from the interpolated response surface which influence the measured and simulated activities. This changes the different GP models and parameter suggestions, especially in the first iterations where most sampled points are far from the optimum and the simulated values are obtained from sparsely populated regions of the design space. With increasing number of iterations, the sampled points are closer to the optimum, where the predictions obtained from the response surface are more accurate. Thus, both the sampled parameter combinations and the enzymatic activities at those points align more closely between experiment and simulation. Due to the discretization of the design space with a step size of 0.2 for pH, the optimum identified in the HTPS with pH 3.5 could not be reached in the optimization. This is important to notice as pH has the strongest influence on the enzymatic activity for UPO‐ABTS and most other tested enzyme‐substrate pairings (Supporting Information S1: Figure S10). However, the optimal conditions are still very similar to those identified in the HTPS for both the experiment and simulation and yield comparable peak activities. In conclusion, the experiments validate the effectiveness of the fine‐tuned BO for autonomous experimental optimization of enzymatic reaction conditions on the SDL.

**Table 5:** Optimal enzymatic reaction conditions determined by the SDL platform in autonomous experiments.

Enzyme	Substrate	pH [‐]	Temperature [°C]	Na_2_SO_4_ [mM]	ACN [%(v/v)]	H_2_O_2_ [mM]
UPO (Exp.)	ABTS	3.4	20	0	0	10.0
UPO (Sim.)	ABTS	3.4	30	125	0	8.0
HRP	ABTS	3.6	28	25	0	2.4
HRP	Pyrogallol	8	20	125	0	10.0
HRP	TMB	4.4	60	175	0	10.0

To test the versatility of the SDL and the fine‐tuned optimization algorithm, the enzymatic reaction conditions of another enzyme, horseradish peroxidase (HRP), with different substrates were autonomously optimized. For HRP‐ABTS, the highest specific enzymatic activity over the iterations is shown in Figure 4b. The highest specific rate is 181.2 ± 18.3 U mg^‐1^ in the initial generation and increases to 918.1 ± 155.7 U mg^‐1^ by cycle four, where it plateaus for two cycles. In cycle seven the overall highest specific rate increases again and reaches an overall best of 1603.9 ± 45.4 U mg^‐1^ by cycle 10. The optimal reaction conditions for HRP‐ABTS identified by the SDL are shown in Table 5. For HRP and ABTS the optimal reaction conditions align closely to those reported in previous studies. Gallati reported interfering effects between pH and ABTS and H_2_O_2_ concentration (Gallati 1979). It was shown that decreasing the ABTS concentration lowers the pH optimum of HRP. In addition, H_2_O_2_ and ABTS show competitive binding to the peroxidase. For 0.5 mM ABTS a pH optimum of 3.8 is reported by Gallati et. al and following the reported trend, for the 0.3 mM ABTS used in this study, the optimal pH should be lower. At 0.25 mM ABTS a *K_m_* = 0.158 mM for H_2_O_2_ is reported, indicating that at 2.4 mM ABTS over 90% of the maximal turnover of H_2_O_2_ is reached. The identified temperature optimum of 28°C is consistent with studies on HRP using phenols and guaiacol as substrates reporting similar values (Lavery et al. 2010). To the best of our knowledge, no prior studies exist on the optimal salt and organic solvent concentrations for this enzyme‐substrate pairing. The results suggest, similar to UPOs, that moderate salt concentrations and the absence of organic solvents maximize enzymatic activity of HRP with ABTS.

For HRP with pyrogallol, the maximal enzymatic activity increases from 118.6 ± 8.1 U mg^‐1^ in the initial cycle to 250.9 ± 27.3 U mg^‐1^ by iteration 3 and then plateaus (Figure 4c). In this study, an optimal pH of 8 for the oxidation of pyrogallol is found (Table 5), while previous studies reported and optimal pH of 7 for phenolic substrates (Lavery et al. 2010; Patel et al. 1997). This discrepancy may be attributed to the strongly increasing autoxidation of pyrogallol in alkaline solutions (Veselinović et al. 2012; Doona and Kustin 1993). The optimal pH identified by the SDL is thus the optimum for the superposition of both the enzymatic and nonenzymatic oxidation of pyrogallol in the investigated pH range. This highlights the importance of carefully selecting the parameter ranges for the optimization process.

Using 3,3,5,5‐tetramethylbenzidine (TMB) as substrate for HRP, the overall highest activity plateaued after two iterations, with minor improvements in iteration 4 and iteration 10. Overall, the activity increased from 603.1 ± 43.9 U mg^‐1^ in the initial generation to 1960.4 ± 14.3 U mg^‐1^ in iteration 10. The optimal pH of 4.4 (Table 5) is in accordance with previous studies which report pH optima between 4.0 and 5.0 for HRP and TMB (Gallati and Pracht 1985). For H_2_O_2_ concentration and temperature, lower values, 1 mM and 20°C–30°C are reported (Gallati and Pracht 1985). However, these values are based on longer incubation times of 30 min. In this study, the initial enzymatic rate in the first 30 s is studied, minimizing the effects of enzyme deactivation while covering the rate‐enhancing effects.

For all enzyme‐substrate pairings, convergence of the BO is indicated by a decreasing posterior variance of the model, inferring a decreasing uncertainty about the objective function (Supporting Information S1: Figure S9). For a CV of 10%–15% in the experiments, a posterior variance of 0.01–0.0225 indicates the model uncertainty equals the noise level, which is the minimum that can be achieved. Values in this range have been achieved for all autonomous optimization runs. A threshold for the posterior variance could thus serve as an important criterion to determine when to terminate the optimization process, indicating that further iterations may yield negligible improvements.

Each iteration in the autonomous optimization cycle, including reagent preparation, dispensing, incubation, measurement, computations and automated setup for the next cycle, takes approximately 75 min. In our current setup, every four cycles require manual replacement of reagent reservoirs to ensure stability of labile compounds, which is performed during regular working hours. As a result, a typical optimization campaign is completed in 2–3 days, despite the total experimental runtime being around 15 h for 12 iterations. Future improvements in automated reagent storage could enable fully continuous optimizations runs within a single day.

In summary, the SDL demonstrated the ability to autonomously and efficiently optimize the enzymatic reaction conditions for multiple enzyme‐substrate pairings. Optimal reaction conditions within the five‐dimensional design space are identified within 11 iterations, often also within less. For example, in the cases of HRP–pyrogallol and HRP–TMB, optimal or near‐optimal conditions were reached within only three experimental cycles. This accelerated convergence can largely be attributed to more favorable initial samples generated by LHS, as the highest activities in these initial sets were already closer to the eventual optimum compared to UPO‐ABTS and HRP‐ABTS, effectively accelerating the optimization process. However, this additionally indicates that the rate of the optimization is still dependent on the initial parameter combinations. To further improve the system, prior knowledge, fo example, from simulations or data from literature or databases could be used to initialize the first set of parameter combinations and further improve performance of the SDL.

The identified enzymatic reaction optima align well with existing studies, highlighting the SDLs reliability, versatility and efficiency for accelerating the optimization of enzymatic reactions. The fine‐tuned BO algorithm, initially tested on a single enzyme‐substrate pairing, proved highly generalizable, reinforcing its utility for similar optimization problems across diverse systems. This demonstrates the effectiveness of the proposed approach for tailoring experimental planning algorithms to improve SDL efficiency.

## 3 Conclusion

In this study, we have demonstrated the development of an autonomous, machine learning‐driven self‐driving laboratory (SDL) platform for the rapid and precise optimization of biochemical reactions. By integrating fully automated experimental workflows, machine learning‐driven optimization, and a seamless Electronic Lab Notebook (ELN)‐connected workflow, the system accelerates traditionally labor‐intensive optimization processes, while being highly user‐friendly. This SDL was applied to optimize enzymatic reaction conditions in a five‐dimensional design space. To further enhance the efficiency of the SDL, we tested and fine‐tuned multiple machine learning algorithms in‐silico, using simulated optimization campaigns on an exemplary data set. Among the tested algorithms, Bayesian Optimization (BO) with a Gaussian Process (GP) model, a Matérn 32 kernel, and an Expected Improvement (EI) acquisition function emerged as the most efficient, achieving superior convergence rates and robust high performance across multiple initial parameter sets.

Real‐world optimization campaigns using the fine‐tuned BO algorithm on the SDL validated its efficiency and generalizability to different enzyme‐substrate pairings. Using the BO, the SDL successfully identified optimal reaction conditions for diverse enzyme‐substrate pairings with a small number of experiments. Importantly, the identified optima aligned well with prior literature, further validating reliability and generalizability of the approach. These results emphasize the platform's potential to accelerate the solving of complex, multi‐parameter optimization challenges across biocatalytic applications. For the best‐performing optimization algorithm identified in the presented work, we expect similar performance for other enzyme–substrate systems and, more generally, for similar biocatalytic optimization problems involving continuous‐valued parameters in moderate to high‐dimensional experimental spaces. The presented SDL platform, as implemented, thus has the potential to accelerate process development in diverse fields of biocatalysis including enzyme‐driven biotransformations, pharmaceutical synthesis, and biosensors. Moreover, the proposed approach for fine‐tuning SDLs with tailored optimization algorithms can be broadly generalized to different fields of application.

While the SDL platform demonstrated robust autonomous optimization capabilities, several challenges remain. Our current system is designed primarily for water‐based enzymatic reactions, as many organic solvents and chemical transformations are not compatible with the current liquid handling hardware. To support a broader chemistry space, additional device modules and safety infrastructure, such as fume hoods, would be required. Manual intervention is still necessary for accurate preparation and placement of reagents and labware. Fully automating these steps through robotic sample preparation and handling would minimize hands‐on labor and improve reproducibility. Handling and storage of labile or temperature‐sensitive reagents currently require manual replacement of reservoirs after several cycles. Automated cooled storage solutions would enable longer, uninterrupted campaigns. Experiment time, reagent consumption, and safety constraints are managed via manual pre‐experiment estimation, and campaign length and parameter boundaries are set accordingly. Future iterations could integrate automated tracking of reagent usage, time, and cost directly into the workflow and optimization logic, allowing for dynamic, constraint‐aware experimentation. Computational scalability is another bottleneck, particularly for Bayesian Optimization as the data set and design space grow. While current computational times, maximally up to a few minutes, are not prohibitive, they are expected to become a limiting factor with larger datasets and higher dimensional design spaces. Algorithmic improvements, GPU acceleration, or distributed computing will be needed for large‐scale applications. Lastly, while the workflow is modular, significant expertise is required for the implementation of new processes.

Looking ahead, integrating large language models (LLMs) into the system is a promising approach to address this challenge while enhancing the SDL's intelligence and versatility. The use of agentic LLMs, AI models equipped with scientific and experimental tools, offers exciting prospects for the field: enabling flexible control of laboratory automation through natural language, automated generation of experimental protocols and device scripts, interactive data analysis and AI‐lead, data‐driven optimization campaigns. Integrating LLMs with the SDL framework has the potential to not only make experimental planning and execution more accessible to a broader user base, but also to accelerate discovery through intelligent hypothesis generation and adaptive experimentation.

In summary, the presented SDL platform establishes an efficient and extensible foundation for accelerated autonomous process optimization and discovery, paving the way for future advancements in biocatalysis and related fields. Ongoing efforts to address current limitations, alongside continued integration of advanced AI technologies, promise to further enhance the platform's efficiency, flexibility, and scientific impact.

## 4 Experimental Section

### 4.1 Materials

Hydrogen peroxide (H_2_O_2,_ 3% (w/w)) and citric acid monohydrate (CA) were purchased from VWR Chemicals (Germany). Horseradish peroxidase Type VI (HRP), di‐sodium hydrogen phosphate (Na_2_HPO_4_), pyrogallol and 3,3,5,5‐tetramethylbenzidine dihydrochloride hydrate (TMB‐d), sodium hydroxide (NaOH, 1.0 N) and hydrochloric acid (HCl, 36.5‐38%) were obtained from Sigma Aldrich (Germany). Dimethyl sulfoxide (DMSO), acetonitrile (ACN), sodium sulfate anhydrous (Na_2_SO_4_), sodium sulfite anhydrous (NaSO_3_) and sulfuric acid (H_2_SO_4,_ 95‐97%) were purchased from Merck (Germany). 2,2‐Azino‐bis(3‐ethylbenzothiazoline‐6‐sulfonic acid) diammonium salt (ABTS) and sodium azide 1% (NaN_3_) were procured from Thermo Fisher Scientific (Germany) and G‐Biosciences (USA). All chemicals and reagents were used as received without additional purification. Buffers were prepared in UPW and pH adjusted using 1 M NaOH and 1 M HCl.

### 4.2 Automated HTPs of Initial Enzymatic Reaction Rates

In the first step, the automated robotic setup of the SDL was employed to generate a comprehensive data set of enzymatic activities, designed for in‐silico testing of various optimization strategies. The initial reaction rate of an unspecific peroxygenase (UPO) with a streptavidin tag (generously provided by Niklas Teetz, BLT, KIT, Germany) was screened using an ABTS assay. This screening was conducted across a range of pH, temperature, and concentrations of salt, organic solvent, and cosubstrate. The colorimetric ABTS assay is based on the principle that the enzymatic oxidation of ABTS produces a green‐colored radical cation (ABTS•⁺), whose formation can be monitored spectrophotometrically at 420 nm. A phosphate‐citrate (PC) buffer, consisting of varying ratios of 0.1 M CA and 0.2 M Na_2_HPO_4_ to adjust pH, was used as buffer system. Na_2_SO_4_ was used as additional salt, ACN as additional organic solvent, and H_2_O_2_ as cosubstrate. The parameter ranges investigated during this study are summarized in Table 6.

**Table 6:** Parameter ranges for the HTPS of initial enzymatic reaction rates of UPO in ABTS assay.

Parameter	Lower limit	Upper limit
pH [−]	2.5	8
Temperature [°C]	20	60
Salt concentration [mM]	0	500
Organic solvent concentration [% (v/v)]	0	30
Cosubstrate concentration [mM]	0	10

To efficiently explore the entire design space with a limited number of experiments, an iterative approach using 3‐Level Full‐Factorial Designs was employed. In the first iteration, the experimental bounds were defined by the predefined upper and lower parameter limits. For the second iteration, the parameter ranges were halved and centered around the best‐performing conditions from the previous iteration—specifically, the experimental setup that achieved the highest initial enzymatic reaction rate. If centering around the optimum led to impractical values (e.g., negative or zero), the center point was repositioned within the new range to ensure feasible experimental conditions. For instance, if a parameter previously ranged from 0 to 100 and the optimal value was found at 0, the next iteration adjusted the range to 0 to 50 instead of extending into negative values (e.g., −50 to 0). The final third iteration was performed analogously again halving the parameter ranges and centering around the updated optimum from the second iteration. All experiments of the screening were conducted as duplicates.

Each iteration of the screening proceeds according to the following workflow. First, during the initialization step the experimental design is generated according to the specified bounds provided in the JSON metadata file. The design is then split into chunks ordered by pH and temperature. Each chunk is randomly shuffled to eliminate any potential bias associated with the timing of experiments. To assess reproducibility and potential instability of reagents, the center point of the design is included in every chunk. For each shuffled chunk of parameter combinations, representing an experimental cycle, control scripts for the pipetting robot (Flex, Opentrons, USA) and the plate reader (Spark, Tecan, Switzerland) are automatically generated before the experiments commence.

Every experimental cycle begins with buffer mixing in 96‐well‐plate format by the pipetting robot to procure the mixtures of the parameter combinations to be tested. The salt concentration is adjusted by mixing phosphate‐citrate (PC) buffers with no additional salt and those with a high salt concentration, both prepared at the desired pH. In the screening, the pH value is adjusted by selecting reservoirs containing buffers at the required pH. The volumes of the cosubstrate, $V_{\mathrm{cosubstrate}}$, and organic solvent, $V_{\mathrm{solvent}}$, are dosed accordingly to adjust the correct concentrations, $c_{\mathrm{cosubstrate}}$ and $c_{\mathrm{solvent}}$, from the provided stock solutions with the concentrations $c_{\mathrm{cosubstrate},\mathrm{stock}}$ and $c_{\mathrm{solvent},\mathrm{stock}}$. The required volumes during this buffer mixing step are calculated according to the following Equations (1) – (4). The total assay volume $V_{\mathrm{total}}$ was fixed at 200 µL. The final concentration, $c_{\mathrm{substrate}}$, and volume, $V_{\mathrm{substrate}}$, of the substrate ABTS were fixed at 0.3 mM and 20 µL, respectively. Stock concentrations of 2 M Na_2_SO_4,_ and 100 mM H_2_O_2_ in PC buffer at the according pH were used. ACN was supplied as pure solvent at 100% (v/v) in the reagent reservoir.

(1) $$V_{\mathrm{buffer},\mathrm{high salt}}=\frac{c_{\mathrm{salt}}}{c_{\mathrm{salt},\mathrm{stock}}}V_{\mathrm{total}}$$

(2) $$V_{\mathrm{cosubstrate}}=\frac{c_{\mathrm{cosubstrate}}}{c_{\mathrm{cosubstrate},\mathrm{stock}}}V_{\mathrm{total}}$$

(3) $$V_{\mathrm{solvent}}=\frac{c_{\mathrm{solvent}}}{c_{\mathrm{solvent},\mathrm{stock}}}V_{\mathrm{total}}$$

(4) $$V_{\mathrm{buffer},\mathrm{low salt}}=V_{\mathrm{total}}-V_{\mathrm{buffer},\;h\mathrm{ig}h\mathrm{salt}}-V_{\mathrm{cosubstrate}}-V_{\mathrm{solvent}}-V_{\mathrm{substrate}}$$

Once the correct volumes of low and high salt buffer, cosubstrate, and solvent are dispensed into the assay well‐plate, the well‐plate is transferred onto the heater‐shaker‐module of the pipetting robot using its internal gripper. The plate is then heated to the target temperature for the assay. After reaching the target temperature, the protocol delays for 5 min to ensure thermal equilibrium between the heating plate and the liquids in the wells. To initiate the enzymatic reaction, substrate and enzyme are dispensed column‐wise into the plate using a multi‐channel pipette. The reaction is allowed to proceed for exactly 60 s before being quenched by the addition of 20 µL of 1% sodium azide. This process is repeated for each column of the well‐plate being tested. After completing the assays, the robotic arm (UR5e, Universal Robots, Denmark), transfers the well‐plate into the plate reader, where the absorbance at 420 nm is measured to quantify the formation of the ABTS•⁺ radical.

Next, the enzymatic reaction rates are calculated according to Equations 5 and 6. First the concentration of the ABTS•⁺ radical is calculated from the raw absorbance data following the Beer–Lambert law (Equation 5):

(5) $$c_{i}=\frac{A_{\lambda ,\mathrm{sample}}-A_{\lambda ,\mathrm{blank}}}{\varepsilon_{i,\lambda }d}$$

Herein, $c_{i}$ is the concentration of the substance i, $A_{\lambda ,\mathrm{sample}}$ the absorbance of the sample at the wavelength λ, $A_{\lambda ,\mathrm{blank}}$ the absorbance of the blank, $\varepsilon_{i,\lambda }$ the extinction coefficient of the substance i at wavelength λ and d the path length. From dividing $c_{i}$ by the reaction time t and the enzyme concentration $c_{\mathrm{enzyme}}$ the specific reaction rate $v_{i,\mathrm{spec}}$ is obtained (Equation 6). Using ABTS and TMB as substrates, the unit (U) of enzymatic activity is defined as the turnover of 1 µmole of substrate per minute. For HRP‐Pyrogallol 1 U oxidizes 1 mg of Pyrogallol in 20 s. In this study, $v_{i,\mathrm{spec}}$ is consequently always presented in U mg^‐1^.

(6) $$v_{i,\mathrm{spec}}=\frac{c_{i}}{tc_{\mathrm{enzyme}}}$$

The data evaluation including calculation of enzymatic reaction rates concludes an experimental cycle and the station prepares for the following cycle by replacing the used well‐plate with a new one from the storage on the work‐bench. Every third cycle, the robotic arm replaces the reagent reservoir in the liquid handler with a new one retrieved from the designated storage positions on the workbench. The system is capable of completing an entire iteration of screening—comprising a 3‐Level Full‐Factorial Design divided in 9 experimental cycles and a total of 513 individual assays—without any human intervention.

### 4.3 Uncertainty Sources and Mitigation Strategies

The SDL system faces several potential sources of experimental uncertainty that must be systematically addressed to ensure reliable optimization results. The liquid handling system was equipped with two pipette types to optimize accuracy across different volume ranges: a Flex 1‐Channel Pipette (1–50 µL) for smaller volumes and a Flex 1‐Channel Pipette (5–1000 µL) for larger volumes (Opentrons Flex 1‐Channel Pipette ‐ Opentrons 2025). For volumes of 20 µL or lower, the smaller volume range pipette was used to minimize error. This dual‐pipette approach minimizes the impact of volume‐dependent errors on the most sensitive assay components. Additionally, stock concentrations of reagents were strategically chosen, so that no pipetting volume was smaller than 5 µL had to be dispensed.

Chemical stability can represent another source of variability in the autonomous system. Enzyme solutions were maintained on ice before addition to assays to prevent thermal deactivation, while liquid reservoirs were sealed with aluminum foil to prevent evaporation and photodegradation. Laboratory lighting and instrument internal lighting were turned off during operations to minimize light‐induced degradation of photosensitive compounds, particularly important for the chromogenic substrates used in the assays. All stock solutions were freshly prepared for each optimization campaign to ensure consistent starting conditions.

Environmental control and temporal factors also contribute to system variability. Temperature and humidity in the laboratory were maintained constant throughout optimization campaigns. During baches, each temperature equilibration step required 4–5 min to ensure thermal stability before reaction initiation, with temperature conditions processed in ascending order to minimize thermal stress on the system. Each experimental cycle required approximately 75 min, during which environmental conditions remained stable. Buffer mixing was completed immediately before temperature equilibration to minimize evaporation and concentration drift that could affect final concentrations.

These mitigation strategies collectively resulted in an average coefficient of variation of 13.2% for the replicates in the HTPS for the ABTS assay, which is consistent with literature values of approximately 12% (Dolz et al. 2023).

### 4.4 In‐Silico Optimization Algorithm Testing in Simulated Experiments

The generated data set for enzymatic reaction rates was used to test and fine‐tune various optimization algorithms in simulated experiments. The identified optimal algorithm finds the optimal reaction conditions, i.e., those that yield the highest reaction rate with the smallest number of iterations, i.e., conducted experiments. Four different types of optimization algorithms were tested: Genetic Algorithm (GA) (Katoch et al. 2021; Vanneschi and Silva 2023), Particle Swarm Optimization (PSO) (Jain et al. 2018; Wang et al. 2018), Simulated Annealing (SA) (Rutenbar 1989; Aarts et al. 2005), and Bayesian Optimization (BO) (Frazier 2018; Shahriari et al. 2016). These algorithms have proven effective for the optimization of continuous variables in diverse biotechnological applications, such enzymatic reactions (Tachibana et al. 2023; Siedentop et al. 2023; Elder et al. 2021; Muffler et al. 2007; Liu et al. 2008) and fermentation processes (Garlapati et al. 2010; Skolpap et al. 2008; Sarma et al. 2009; Subba Rao et al. 2008; Nagata and Chu 2003; Parkhey et al. 2017; Dhagat and Jujjavarapu 2021). Additionally, the algorithm performance was compared to RSM, the most established optimization method in the field of biocatalysis (Aktaş 2005; Lee et al. 2006; Mushtaq et al. 2015; Tacias‐Pascacio et al. 2019). RS was used as a baseline to benchmark the performance of guided optimization algorithms against brute‐force, unguided sampling of the design space.

For each algorithm, key hyperparameters were defined and systematically tested across a range of values. In this context, hyperparameters refer to the parameters affecting the algorithm itself, for example, the mutation rate in a GA. All possible permutations of these hyperparameter values (hereafter referred to as algorithm variants) were evaluated using a grid search approach.

All optimization algorithms were implemented in‐house using Python, relying only on standard scientific libraries for data handling and modeling. For BO, Gaussian process regression was implemented using the *scikit‐learn* library, with the kernel function selected from the available options, default zero mean prior, and the noise level set to 0.02 to reflect the average coefficient of variation (CV) of 13.2% observed in experimental measurements. Hyperparameters were optimized by log marginal likelihood with ten random restarts, and standard acquisition functions were employed with typical literature values. While this homoscedastic noise assumption simplified the modeling, we acknowledge that more advanced heteroscedastic noise modeling (using measured experimental uncertainties) could further improve predictive accuracy in future studies. Detailed descriptions of the optimization algorithms are available in the *Supporting Information, Section 2* and in the published repository (putzsebastian 2025).

**Figure 5:**
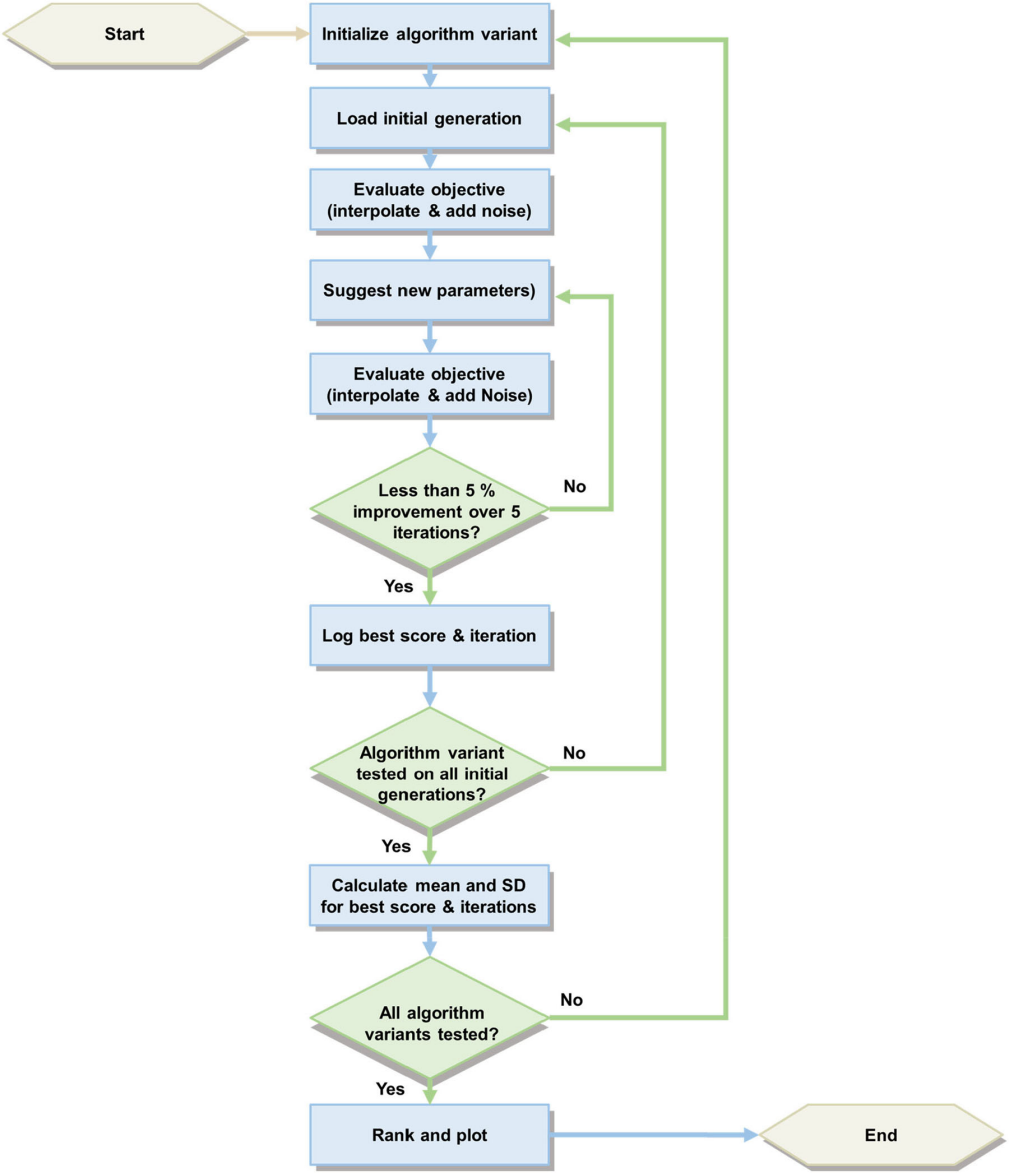
Flow‐chart of the in‐silico optimization algorithm screening.

The testing process was standardized to mimic the cyclic experimental workflow of a self‐driving lab. Each algorithm was initialized with the same 30 unique sets of initial parameter combinations, with each set containing 8 parameter combinations generated via LHS. In the simulated experiments, the enzymatic reaction rate for each parameter combination was calculated using linear interpolation based on the data set. To simulate real‐world experimental noise, a uniform noise in the range of 13.2%, corresponding to the average coefficient of variation (CV) observed during HTPS, was added to the calculated rates. After the enzymatic reaction rates are calculated for the initial generation, the algorithm variant suggests 8 new parameter combinations that are tested in the next round. The best target value, that is, the highest enzymatic reaction rate, in each iteration is tracked. A lack‐of‐improvement criterion was selected to determine convergence of the algorithm. If the best target value did not improve by at least 5% over the last 5 iterations, the optimization process was terminated. The highest enzymatic reaction rate identified and the number of iterations until convergence were recorded. This process was repeated for all 30 initial parameter sets for each algorithm variant. As metrics for comparing different algorithms, the mean and standard deviation of the highest enzymatic reaction rate and the number of iterations until convergence, were calculated across the 30 runs for each algorithm variant. The workflow for this screening of algorithm variants is illustrated as flow‐chart in Figure 5.

### 4.5 Autonomous Optimization of Enzymatic Reaction Conditions in Real Experiments

To validate the best‐performing in‐silico algorithm in real experiments, autonomous optimization campaigns were conducted using the SDL. The platform was utilized to optimize the reaction conditions for the enzyme HRP using the substrates ABTS, Pyrogallol and TMB‐d. Additionally, the reaction conditions for the UPO‐ABTS pairing were optimized to compare them to a simulated optimization run. The optimization aimed to maximize the initial enzymatic reaction rate by varying pH, temperature, and the concentrations of salt, organic solvent, and cosubstrate.

Similar to the screening, a phosphate‐citrate (PC) buffer system, consisting of varying ratios of 0.1 M CA and 0.2 M Na_2_HPO_4,_ was used. Na_2_SO_4_ was used as additional salt, ACN as additional organic solvent and H_2_O_2_ as cosubstrate. The parameter ranges remained consistent with those previously outlined in Table 6. However, for practicality and computational efficiency, the design space was discretized into manageable increments, see Table 7. To accommodate this discretization, the BO was adapted to operate directly on the discrete parameter grid, with acquisition functions evaluated at each grid point rather than through continuous optimization followed by rounding. This approach ensures all proposed parameter combinations are experimentally feasible while avoiding potential artifacts from continuous‐to‐discrete parameter mapping.

**Table 7:** Parameter ranges for the HTPS of initial enzymatic reaction rates of UPO enzyme in ABTS assay.

Parameter	Lower limit	Upper limit	Step size
pH [−]	2.4	8	0.2
Temperature [°C]	20	60	2
Salt concentration [mM]	0	500	25
Organic solvent concentration [% (v/v)]	0	30	2.5
Cosubstrate concentration [mM]	0	10	0.2

To enable testing of different pH values and temperatures within the same experimental cycle, the protocol differed slightly from the automated HTPS experiments. For the first experimental cycle, 8 parameter combinations were generated using LHS. During each cycle, buffers were prepared according to the tested parameter combinations using Equations (1)–(4). However, to adjust pH and salt concentrations without pre‐mixed buffers, the buffer volumes $V_{\mathrm{buffer},\mathrm{high salt}}$ and $V_{\mathrm{buffer},\mathrm{low salt}}$ were further split into separate volumes of 0.1 M CA and 0.2 M Na_2_HPO_4_ with no additional Na_2_SO_4_ and 1 M additional Na_2_SO_4_, according to the mixing table for the PC buffer (McIlvaine 1921). After dosing the required volumes of CA, CA with added salt, Na_2_HPO_4_, Na_2_HPO_4_ with added salt, 100 mM H_2_O_2_ in UPW and ACN (100% (v/v)) were dispensed at according volumes to adjust the correct concentrations of cosubstrate and organic solvent. Then the buffer mixing was complete and the well‐plate was heated to the required temperatures specified in the tested parameter combinations, processed in ascending order. Once the lowest target temperature was reached and stabilized, the pipetting robot dispensed 20 µL substrate and 20 µL enzyme solution (in UPW) into the wells corresponding to that temperature to initiate the reaction. The reaction was stopped at the specified reaction time for each assay. This process was repeated for all temperature levels until all assays for that experimental cycle were completed. The assay‐specific differences are shown in Table 8.

**Table 8:** Assay‐specific parameters for ABTS, Pyrogallol and TMB‐d assay with the enzymes HRP and UPO.

Enzyme	UPO	HRP	HRP	HRP
Substrate	ABTS	ABTS	Pyrogallol	TMB‐d
Substrate concentration	0.3 mM	0.3 mM	40 mM	1 mM
Substrate solvent	UPW	UPW	UPW	DMSO
Enzyme concentration [mg mL^‐1^]	5 × 10^‐4^	5 × 10^‐4^	2.5 × 10^‐3^	1 × 10^‐4^
Reaction time [s]	60	60	60	30
Stop reagent	Sodium azide (1%)	Sodium azide (1%)	Sodium azide (1%) + 1 M NaSO_3_	H_2_SO_4_ (2 M)
Stop reagent volume [µL]	20	20	20	50
Measurement wavelength [nm]	420	420	420	450

After completing the assays, the robotic arm transferred the well‐plate to the plate reader for absorbance measurements. The well‐plate was then returned to the liquid‐handling station. Every fourth cycle, the well‐plate and reagent reservoirs were replaced with fresh ones from the storage on the workbench. Reaction rates were calculated at the end of the cycle using Equations 5 and 6.

The BO algorithm then determined the 8 parameter combinations that are tested in the subsequent experiment cycle. In brief, first, a GP model with a Matérn32 kernel as covariance function was fitted to all thus far generated data points. Using the EI acquisition function, the parameter combination with the highest EI was chosen as candidate for the next cycle. For batch‐wise optimization with multiple parameter combinations per cycle, the Kriging‐believer algorithm was employed to generate a batch of 8 parameter combinations. To avoid redundancy, a feature was implemented so that no parameter combination was tested more than once across all experimental cycles. Details on the utilized BO and all other tested optimization algorithms can be found in the *Supporting Information*, *Section* *2*.

## Author Contributions

**Sebastian Putz:** conceptualization, methodology, experimental work, data analysis, visualization, writing – original draft. **Niklas Teetz**, **Michael Abt:** conceptualization, resources (enzyme material), writing – review and editing. **Pascal Jerono**, **Thomas Meurer:** methodology, writing – review and editing. **Matthias Franzreb:** conceptualization, supervision, project administration, funding acquisition, writing ‐ review and editing.

## Conflicts of Interest

The authors declare no conflicts of interest.

## Supporting information


**Figure S1:** Workflow control panel in the GUI of the SDL software. The GUI was created using the package customtkinter in Python 3. **Figure S2:** Detailed overview of the SDL software showing software modules, repositories, devices, utilized and created files. **Figure S3:** Flow‐chart for the utilized genetic algorithm (GA). **Figure S4:** Flow‐chart for the utilized Particle Swarm Optimization (PSO) algorithm. **Figure S5:** Flow‐chart of the utilized Bayesian Optimization (BO) algorithm. **Figure S6:** Flow‐chart of the utilized Simulated Annealing (SA) algorithm. **Figure S7:** Flow‐chart of the utilized random search (RS) algorithm. **Figure S8:** Flow‐chart of the Response Surface Modelling (RSM). **Figure S9:** Posterior variance in the autonomous enzymatic reaction condition optimization experiments on the SDL for the enzyme‐substrate‐pairings (a) UPO‐ABTS, (b) HRP‐ABTS, (c) HRP‐Pyrogallol and (d) HRP‐TMB. **Figure S10:** Parameter importances determined by Random Forest Regression in the autonomous enzymatic reaction condition optimization experiments on the SDL for the enzyme‐substrate‐pairings (a) UPO‐ABTS, (b) HRP‐ABTS, (c) HRP‐Pyrogallol and (d) HRP‐TMB. **Figure S11:** Visualization of the linear interpolation surrogate model and uncertainty proxies for the enzymatic activity landscape as a function of pH and temperature (other parameters fixed at optimal values: cH_2_O_2_ = 8.75 mM, cNa_2_SO_4_ = 120 mM, c_ACN_ = 0% v/v). **Figure S12:** Robustness analysis of the interpolated landscape to experimental noise (±1 SD). Six independent realizations (a)–(f) of the interpolated mean activity surface as a function of pH and temperature (other parameters fixed at optimal values: cH_2_O_2_ = 8.75 mM, cNa_2_SO_4_ = 120 mM, c_ACN_ = 0% v/v) were generated by adding random noise within ±1 standard deviation at each grid point. **Figure S13:** Robustness analysis of the interpolated landscape to increased experimental noise (±2 SD). Six independent realizations (a)–(f) of the interpolated mean activity surface as a function of pH and temperature (other parameters fixed at optimal values: cH_2_O_2_ = 8.75 mM, cNa_2_SO_4_ = 120 mM, c_ACN_ = 0% v/v) were generated by adding random noise within ±2 standard deviation at each grid point. **Table S1:** Tested hyperparameters of the genetic algorithm (GA) in the in‐silico algorithm screening. **Table S2:** Tested hyperparameters of the Particle Swarm Optimization (PSO) algorithm in the in‐silico algorithm screening. **Table S3:** Tested hyperparameters of the Bayesian Optimization (BO) algorithm in the in‐silico algorithm screening. **Table S4:** Tested hyperparameters of the Simulated Annealing (SA) algorithm in the in‐silico algorithm screening.

## Data Availability

The primary data set and codebase that support the findings of this study are openly available in GitHub under https://github.com/putzsebastian/sdl-enzymes-optimization (https://doi.org/10.5281/zenodo.15730516). Additional data and code are available from the corresponding author upon reasonable request.
