# Supplementary material for: Optimized Machine Learning for Autonomous Enzymatic Reaction Intensification in a Self‐Driving Lab
Source: Biotechnol Bioeng. 2025 Aug 4;122(11):3018–36. doi: 10.1002/bit.70038 (PMC12503018; doi:10.1002/bit.70038)
Supplement: Supplementary file 1 — Figure S1: Workflow control panel in the GUI of the SDL software. The GUI was created using the package customtkinter in Python 3. Figure S2: Detailed overview of the SDL software showing software modules, repositories, devices, utilized and created files. Figure S3: Flow‐chart for the utilized genetic algorithm (GA). Figure S4: Flow‐chart for the utilized Particle Swarm Optimization (PSO) algorithm. Figure S5: Flow‐chart of the utilized Bayesian Optimization (BO) algorithm. Figure S6: Flow‐chart of the utilized Simulated Annealing (SA) algorithm. Figure S7: Flow‐chart of the utilized random search (RS) algorithm. Figure S8: Flow‐chart of the Response Surface Modelling (RSM). Figure S9: Posterior variance in the autonomous enzymatic reaction condition optimization experiments on the SDL for the enzyme‐substrate‐pairings (a) UPO‐ABTS, (b) HRP‐ABTS, (c) HRP‐Pyrogallol and (d) HRP‐TMB. Figure S10: Parameter importances determined by Random Forest Regression in the autonomous enzymatic reaction condition optimization experiments on the SDL for the enzyme‐substrate‐pairings (a) UPO‐ABTS, (b) HRP‐ABTS, (c) HRP‐Pyrogallol and (d) HRP‐TMB. Figure S11: Visualization of the linear interpolation surrogate model and uncertainty proxies for the enzymatic activity landscape as a function of pH and temperature (other parameters fixed at optimal values: cH2O2 = 8.75 mM, cNa2SO4 = 120 mM, cACN = 0% v/v). Figure S12: Robustness analysis of the interpolated landscape to experimental noise (±1 SD). Six independent realizations (a)–(f) of the interpolated mean activity surface as a function of pH and temperature (other parameters fixed at optimal values: cH2O2 = 8.75 mM, cNa2SO4 = 120 mM, cACN = 0% v/v) were generated by adding random noise within ±1 standard deviation at each grid point. Figure S13: Robustness analysis of the interpolated landscape to increased experimental noise (±2 SD). Six independent realizations (a)–(f) of the interpolated mean activity surface as a function of pH [file BIT-122-3018-s001.docx]

Supporting Information

Optimized Machine Learning for Autonomous Enzymatic Reaction Intensification in a Self-driving Lab

Sebastian Putz, Niklas Teetz, Michael Abt, Pascal Jerono, Thomas Meurer, Matthias Franzreb*

1. **Details on the SDL software**

The SDL is controlled by a modular software framework written in Python 3. The individual modules are shown in Figure S2. After designing the experiment in eLabFTW on the basis of a template, the user interacts with the SDL using a simple custom GUI (Figure S1). Before accessing the workflow control panel, the user must login using his eLabFTW user and experiment ID as well as a password. Using the GUI, the user navigates the workflow by simply clicking the buttons in order. The only inputs required are the device IPs in case of change.

**
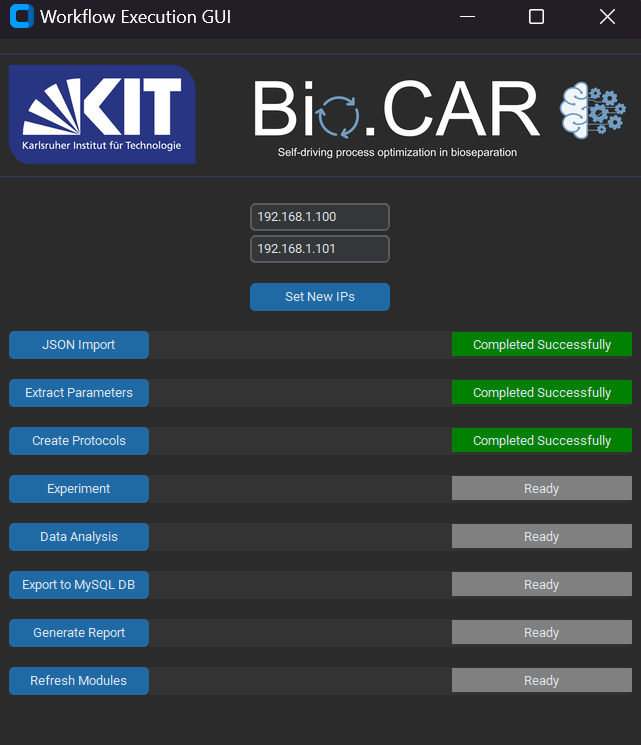
**

**Figure S1:** Workflow control panel in the GUI of the SDL software. The GUI was created using the package customtkinter in Python 3.

**
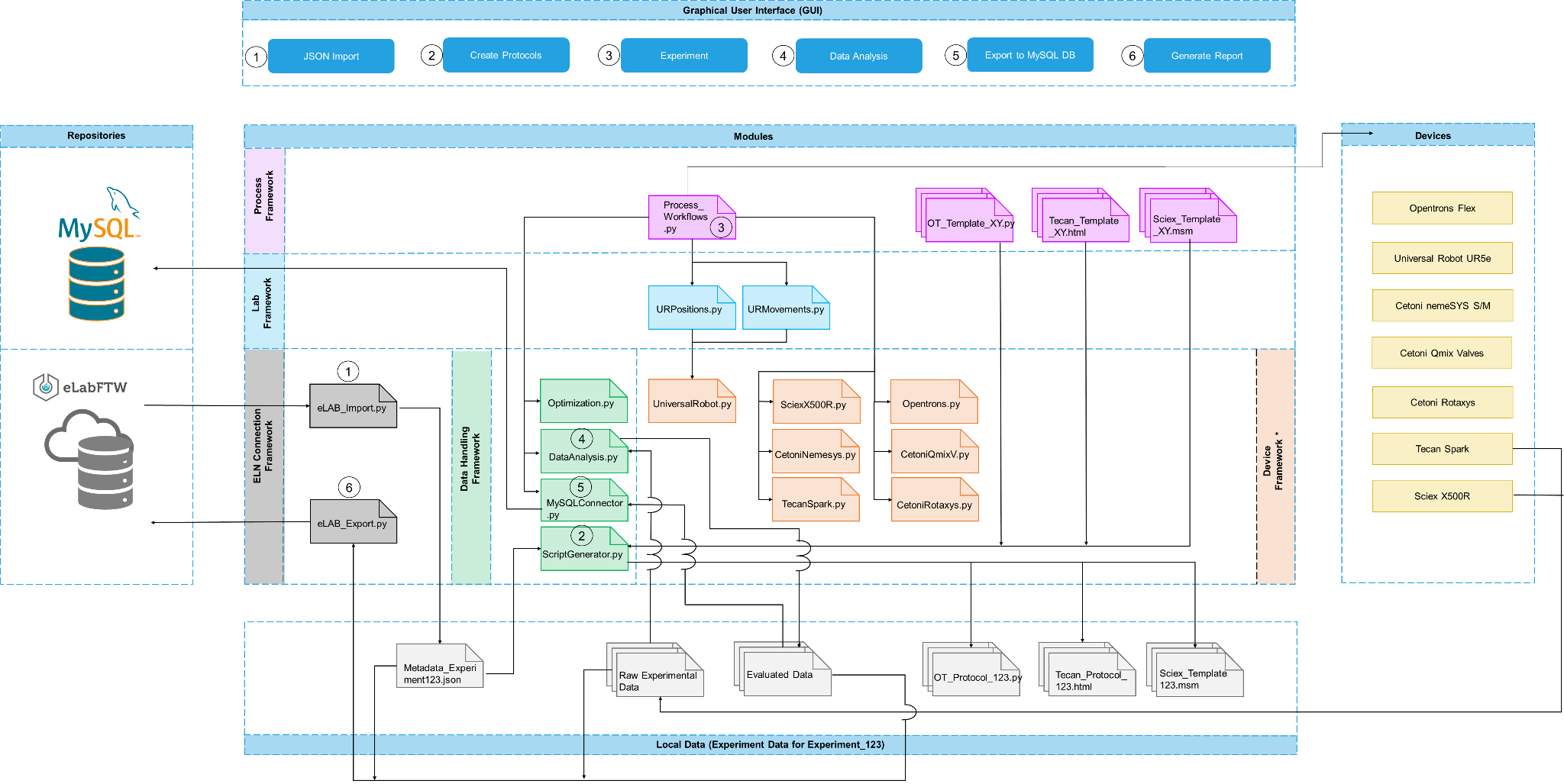
**

**Figure S2:** Detailed overview of the SDL software showing software modules, repositories, devices, utilized and created files.

1. **Utilized optimization algorithms**

**2.1. Genetic Algorithm (GA)**

A genetic algorithm (GA) is an optimization method inspired by the process of natural selection in biological systems. The flowchart (Figure S3) represents the key steps of the utilized genetic algorithm, which are outlined below.

1. Initialization

- In the initialization step, the first generation of candidate solutions is either created randomly or by a sampling method. For the in-silico algorithm testing, the initial generations are loaded from a predefined set of parameter combinations. Each individual represents a set of enzyme reaction parameters (pH, salt concentration, cosubstrate concentration, organic solvent concentration, and temperature)
- These initial guesses serve as the starting point for the optimization process.

2. Evaluation

- The algorithm runs the process with these initial parameters and evaluates their performance.
- Each candidate solution in the population is evaluated based on a fitness function, which measures how well a particular solution performs in relation to the optimization objective. For the in-silico algorithm testing, the fitness function is the specific enzymatic activity. The activity is calculated by linear interpolation between the existing datapoints in the dataset. This allows for the calculation of fitness values in the complete parameter space without introducing additional bias into the fitness function, compared to fitting a predetermined model to the data.

3. Selection

- The selection step involves choosing the best-performing solutions (parents) from the current population for creating the next generation.
- Various selection methods can be used, such as roulette wheel selection, tournament selection, or rank-based selection. With either selection method 4 individuals are selected as parents for the next round:
  - Roulette wheel selection: In roulette wheel selection, individuals are selected with a probability proportional to their fitness. The method can be imagined as a roulette wheel where each individual is assigned a segment of the wheel proportional to its fitness. Higher fitness individuals have larger segments, giving them a higher chance of being selected. A random spin of the wheel determines which individual is selected.
  - Tournament selection: a fixed number of individuals (in this case 3) are randomly chosen from the population. The individual with the highest fitness within the selected group is chosen as a parent. This process is repeated until the desired number of parents is selected.
  - Rank-based selection: All individuals in the population are ranked based on their fitness values, with the best individual given the highest rank and the worst the lowest. The highest-ranked individuals are chosen as parents for the next generation.
  - The goal for all selection methods is to give higher fitness solutions a greater chance of passing their traits to the next generation.
- If elitism is employed, a specified number of top ranked individuals are carried over into the next generation without any alteration or mutation. In this study 0, 1 or 2 top-performing individuals are used with this feature.

4. Recombination

- To create the remaining individuals for the next generation, recombination (or crossover) is performed by combining parts of two or more selected parent solutions.
- The offspring inherit characteristics from their parents, with the goal of producing new solutions that may perform better than the current ones.
- Different recombination techniques were used, namely single-point, random and uniform crossover. Two parents are randomly chosen from the 4 selected parents for each recombination method:
  - Single-point: A random crossover point is selected along the length of the parents. The first part of *parent1* (up to the crossover point) is combined with the second part of *parent2* to create *child1.* Similarly, the first part of *parent2* is combined with the second part of *parent1* to create *child2*.
  - Uniform: A binary mask of the same length as the parents is generated randomly, where each element is either *True* or *False* with equal probability. *child1* is formed by taking elements from *parent1* wherever the mask is *True* and from *parent2* wherever the mask is *False.* *child2* is formed in the reverse manner.
  - Random: Instead of using a specific crossover point or a mask, this method randomly selects a value for each position from either *parent1* or *parent2.*

5. Mutation

- Mutation introduces random variations to the offspring by altering one or more of their parameters.
- This step ensures genetic diversity in the population and helps the algorithm explore a broader search space, preventing it from getting stuck in local optima.
- For each individual in the next generation, after the cross-over step, each parameter can mutate with a certain mutation probability which determines the likelihood that a given parameter will undergo mutation.
- The mutation extent determines the magnitude of the change applied when a mutation occurs. It controls how far a parameter is allowed to deviate from its original value. It is defined as a percentage of the parameter range.

6. Evaluation & Ranking (Repeated)

- The new generation of solutions created through recombination and mutation is evaluated and ranked using the same fitness function.
- The best solutions from this new population are selected again, continuing the evolutionary process.

7. Stopping Criterion Check

- After each iteration (generation), the algorithm checks whether a predefined stopping criterion has been met.
- In the in-silico algorithm testing, this is defined as the lack of significant improvement in fitness values over several generations. Specifically, an improvement of less than 5% of the overall best solution across 5 consecutive iterations triggers termination.
- If the stopping criterion is met, the algorithm terminates, and the best solution is returned as the final result.
- If not, the algorithm repeats the process from the selection step with the new population.

Evaluated Hyperparameters

For the GA, 5 hyperparameters, namely the selection method, the cross-over method, the elitism, the mutation probability and mutation extent were varied in a full grid-search during the screening for the best performing algorithm variant. The hyperparameters and the tested values are shown in Table S1.


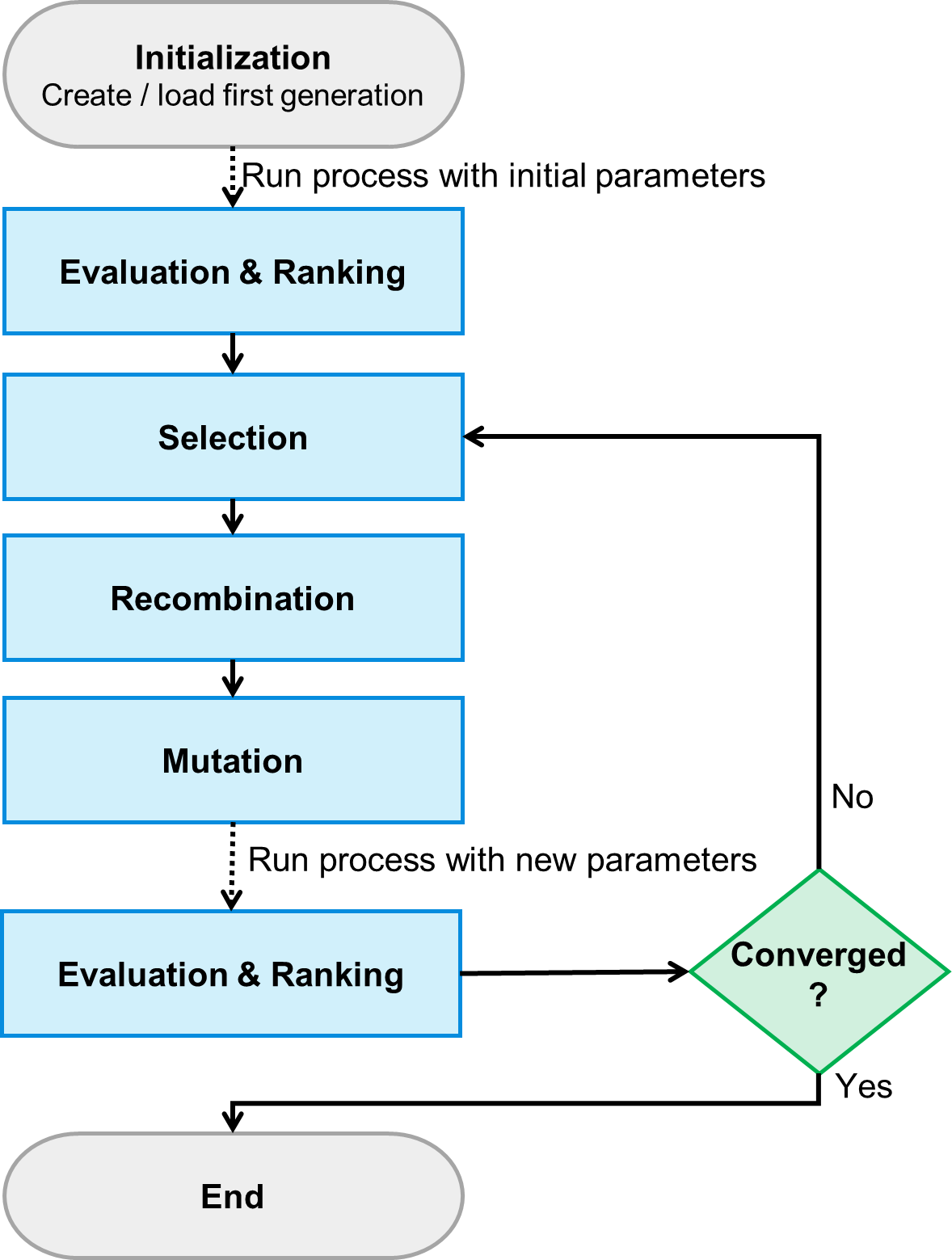
**Figure S3:** Flow-chart for the utilized genetic algorithm (GA).

**Table S1:** Tested hyperparameters of the genetic algorithm (GA) in the in-silico algorithm screening.

| **Hyperparameter** | **Tested values** |
| --- | --- |
| Selection method | Rank, tournament, roulette |
| Cross-over method | Random, single-point, uniform |
| Elitism | 0, 1, 2 |
| Mutation Probability | 0.1, 0.2, 0.4, 0.8 |
| Mutation Extent | 0.1, 0.3, 0.6, 1 |

**2.2 Particle Swarm Optimization (PSO)**

The particle swarm optimization (PSO) algorithm is a population-based stochastic optimization technique inspired by the social behavior of birds flocking or fish schooling. In this implementation, PSO is applied to optimize enzyme reaction conditions based on interpolated or extrapolated experimental data. The key steps of the PSO are shown in Figure S4 and explained in the following:

**1. Initialization**

- At the beginning, a swarm of particles (candidate solutions) is initialized. Each particle represents a set of enzyme reaction parameters (pH, salt concentration, cosubstrate concentration, organic solvent concentration, and temperature).
- The particles are assigned random initial velocities, and their initial positions are loaded from predefined combinations generated using Latin Hypercube Sampling (LHS) to ensure a well-distributed search of the parameter space.
- Each particle starts with an undefined personal best solution (*p_Best_*), while a global best solution (*g_Best_*) is initialized from one of the particles.

**2. Objective Function Evaluation**

- In each iteration, the objective function is evaluated for all particles to determine the fitness of their current positions. The objective function involves linear interpolation of the specific activity based on a pre-existing dataset of enzyme reaction results.

**3.** Update of Personal Best (*p_Best_*)

- If a particle's current fitness is better than its previously recorded personal best fitness, the personal best position and fitness are updated accordingly.

**4. Update of Global Best (***g_Best_***)**

- After updating the personal bests, the algorithm checks whether any particle has achieved a fitness better than the current global best solution. If such a particle is found, the global best position and fitness are updated. This ensures that the swarm collectively moves towards the most promising region of the search space.

**5. Velocity Update**

- The velocity of each particle is updated based on three components:
  - Inertia: This component retains part of the particle's current velocity, encouraging it to continue in its current direction.
  - Cognitive Component: This term pulls the particle towards its personal best position, encouraging self-exploration.
  - Social Component: This term pulls the particle towards the global best position, promoting cooperation among particles.
- The influence of each component is controlled by three parameters: the inertia weight ($w$), the cognitive coefficient ($c_{1}$), and the social coefficient ($c_{2}$). The inertia weight decreases exponentially, with a constant decay factor $c_{d}$ ϵ (0,1) (Eq. S1):

| $w^{(t+1)}=w^{(t)} c_{d}$ | (S1) |
| --- | --- |

- Random factors, $r_{1}$ and $r_{2}$ ϵ (0,1), are introduced to the cognitive and social components to add stochasticity and ensure diverse exploration (Eq. S2).

| $v_{i}^{(t+1)}=w^{(t+1)}v_{i}^{(t)}+ c_{1}r_{1}\left( p_{Best}-p_{i}^{t} \right)+ c_{2}r_{2}\left( g_{Best}-p_{i}^{t} \right)$ | (S2) |
| --- | --- |

**6. Position Update**

- Once the new velocities are computed, they are used to update the positions of the particles. Each particle’s position is adjusted by adding its corresponding velocity (Eq. S3).

| $p_{i}^{(t+1)}=p_{i}^{(t)}+ v_{i}^{(t+1)}$ | (S3) |
| --- | --- |

**7. Stopping Criterion Check**

- After each iteration, the algorithm checks whether a stopping criterion has been met.
- The stopping criterion was defined as a lack of significant improvement in fitness values over several generations. As threshold an improvement of 5 % of the overall best solution over 5 iterations was selected.
- If the stopping criterion is met, the algorithm terminates, and the best solution is returned as the final result.
- If not, the algorithm repeats the process from the selection step with the new solutions updating personal and global bests, and computing new velocities and positions. Otherwise, it terminates, returning the global best solution found during the optimization process.

**Evaluated Hyperparameters:**

For the PSO, 4 hyperparameters, namely the initial inertia weight $w_{0}$, the inertia decay factor $c_{d}$, the cognitive coefficient $c_{1}$and the social coefficient $c_{2}$ were varied (Table S2).

**
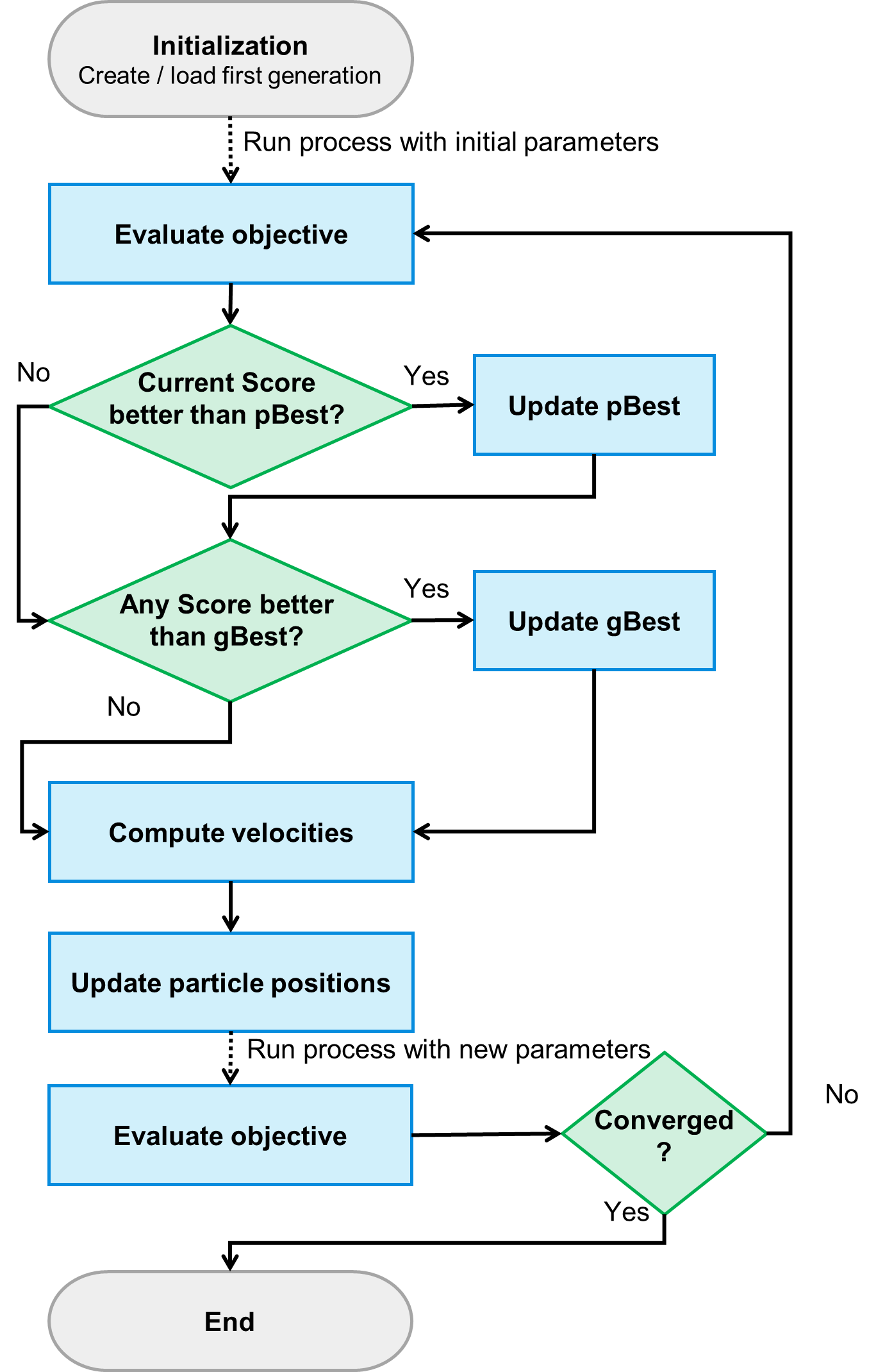
**

**Figure S4:** Flow-chart for the utilized Particle Swarm Optimization (PSO) algorithm.

**Table S2:** Tested hyperparameters of the Particle Swarm Optimization (PSO) algorithm in the in-silico algorithm screening.

| **Hyperparameter** | **Tested values** |
| --- | --- |
| $w_{0}$ | 0.5, 0.7, 0.9, 1.1 |
| $c_{d}$ | 0.65, 0.75, 0.9, 1.1 |
| $c_{1}$ | 1.0, 1.5, 2.0, 2.5 |
| $c_{2}$ | 1.0, 1.5, 2.0, 2.5 |

**2.3. Bayesian Optimization BO**

Bayesian Optimization is a sequential optimization strategy that aims to find the global optimum of an objective function with minimal evaluations. Unlike traditional optimization methods, it uses a probabilistic surrogate model, typically a Gaussian Process (GP), to approximate the objective function and an acquisition function to balance exploration of uncertain regions and exploitation of promising areas. By iteratively updating the surrogate model and selecting new candidates based on maximizing the acquisition function, Bayesian Optimization efficiently identifies optimal solutions with minimal function evaluations. This approach is particularly advantageous in scenarios where each evaluation is costly, as it significantly reduces the required number of experiments while maximizing performance.

**1. Initialization**

- In the initialization step, the first generation of candidate solutions is either created randomly or by a sampling method. For the in-silico algorithm testing, the initial generations are loaded from a predefined set of parameter combinations. Each initial solution is represented by a set of enzyme reaction parameters (pH, salt concentration, cosubstrate concentration, organic solvent concentration, and temperature)
- These initial solutions serve as the starting point for the optimization process.

**2. Evaluate Objective Function**

- In each iteration, the objective function is evaluated for all particles to determine the fitness of their current positions. The objective function involves interpolation of the specific activity based on a pre-existing dataset of enzyme reaction results. These scores serve as the basis for training the probabilistic surrogate model.

**3. Fit Probabilistic Surrogate Model**

- A probabilistic surrogate model, specifically a Gaussian Process Regressor, is fitted to the evaluated data. This model estimates the objective function over the entire parameter space, including uncertainty in the predictions. A Gaussian Process is a collection of random variables, any finite number of which follows a multivariate normal distribution. In the context of BO, the GP defines a distribution over possible objective functions (Eq. S4).

| $f\left( x \right) \sim GP(\mu\left( x \right), k\left( x, x^{'} \right))$ | (S4) |
| --- | --- |

- Herein, $\mu\left( x \right)$ is the mean function and $k\left( x, x^{'} \right)$ is the **kernel function** (or covariance function), which defines the similarity between two points $x$ and $x^{'}$.
- The model is trained using the standardized versions of the input parameters and their corresponding objective values. Different kernels, such as Matérn, RBF, and Rational Quadratic, can be used to control the smoothness and flexibility of the model. In Eqs. S5-S7, $l$ is the length scale parameter, with $l>0$. The length scale parameter in Bayesian Optimization (BO) kernel functions determines how quickly correlations between data points decay as their distance increases in the input space, thereby controlling the smoothness and flexibility of the surrogate model. In the rational quadratic kernel, the $\alpha$parameter, with $\alpha>0,$ controls the relative weighting between large-scale and small-scale variations, allowing the kernel to interpolate between the behavior of a squared exponential kernel and a sum of many kernels with different length scales.
  - Radial Basis Function (RBF): $k\left( x,x^{'} \right)=exp\left( -\frac{{|x-x^{'}|}^{2}}{2l^{2}} \right)$ (S5)
  - Rational Quadratic (RQ): $k\left( x,x^{'} \right)=exp\left( 1+\frac{{|x-x^{'}|}^{2}}{2\alpha l^{2}} \right)^{-\alpha}$ (S6)
  - Matérn 1/2: $k\left( x,x^{'} \right)=exp\left( -\frac{|x-x^{'}|}{l} \right)$ (S7)
  - Matérn 3/2: $k\left( x,x^{'} \right)=\left( 1+\frac{\sqrt{3}|x-x^{'}|}{l} \right)exp\left( -\frac{\sqrt{3}|x-x^{'}|}{l} \right)$ (S8)
  - Matérn 5/2: $k\left( x,x^{'} \right)=\left( 1+\frac{\sqrt{5}|x-x^{'}|}{l}+\frac{\sqrt{5}|{x-x^{'}|}^{2}}{3l^{2}} \right)exp\left( -\frac{\sqrt{5}|x-x^{'}|}{l} \right)$ (S9)

**4. Maximize Acquisition Function**

The algorithm then uses an acquisition function to identify new candidate parameter sets. The acquisition function balances exploration (searching areas with high uncertainty) and exploitation (searching areas with high predicted values). Several acquisition functions are used, including:

- **Expected Improvement (EI):** Prefers areas where the predicted improvement over the current best solution $x^{+}$ is highest (Eq. S10).

$EI\left( x \right)=\left( \mu_{n}\left( x \right)-f\left( x^{+} \right) \right)\Phi\left( Z \right)+\sigma_{n}(x)\varphi(Z)$ (S10)

Herein,

- $\mu_{n}\left( x \right)$ **is the predicted mean of the Gaussian Process at** $x$
- $\sigma_{n}(x)$ **is the predicted standard deviation of the Gaussian Process at** $x$
- $f\left( x^{+} \right)$ **is the best observed objective value so far (i.e., the highest or lowest, depending on whether it’s a maximization or minimization problem).**
- $Z$ **is a standard normal variable defined as:**

$Z=\frac{\mu_{n}\left( x \right)-f\left( x^{+} \right)}{\sigma_{n}(x)}$ (S11)

- $\Phi\left( Z \right)$ **is the cumulative distribution function (CDF) of the standard normal distribution.**
- $\varphi(Z)$is the probability density function (PDF) of the standard normal distribution.
- **Probability of Improvement (PI):** Selects areas with the highest probability of improving the current best solution (Eq. S12).

$PI\left( x \right)= \Phi\left( Z(x) \right)$ (S12)

- **Upper Confidence Bound (UCB):** Balances exploration and exploitation by considering both the predicted mean and uncertainty (Eq. S13).

$UCB\left( x \right)= \mu_{n}\left( x \right)+\kappa\sigma_{n}(x)$, $\kappa>0$ (S13)

- **Epsilon-Greedy Strategy:** Chooses $x^{*}$ randomly from the uniform distribution $\mathcal{U}\left( \mathcal{X} \right)$ of the design space $\mathcal{X}$ with probability $\varepsilon$ or the point with the highest predicted mean $\mu(x)$ with probability 1 – $\varepsilon$ (Eq. S14).

$x^{*}=\left\{ \begin{aligned} x\sim\mathcal{U}\left( \mathcal{X} \right) with probability \varepsilon\\ \arg\max\mu\left( x \right) with probability 1-\varepsilon\end{aligned} \right.$ (S14)

**5. Batchwise Candidate Points Selection**

- **For batch-wise selection of candidate points for the next iteration, the Kriging Believer** algorithm is employed. This method sequentially selects new points by assuming that previously selected points will yield the predicted mean.

**6. Evaluate Objective Function for New Points**

- The newly suggested parameter sets are evaluated using the objective function, and their scores are added to the dataset. This updated dataset is then used to retrain the Gaussian Process model in the next iteration.

**7. Convergence Check**

- After each iteration, the algorithm checks whether a stopping criterion has been met.
- The stopping criterion was defined as a lack of significant improvement in fitness values over several generations. As threshold an improvement of 5 % of the overall best solution over 5 iterations was selected.
- If the stopping criterion is met, the algorithm terminates, and the best solution is returned as the final result.
- Otherwise, the algorithm proceeds to the next iteration by updating the surrogate model with newly acquired data points, recalculating the acquisition function, and selecting the next candidate points for evaluation.

**Evaluated Hyperparameters:**

For the BO, 2 hyperparameters, namely the kernel function and the acquisition function were varied (Table S3).


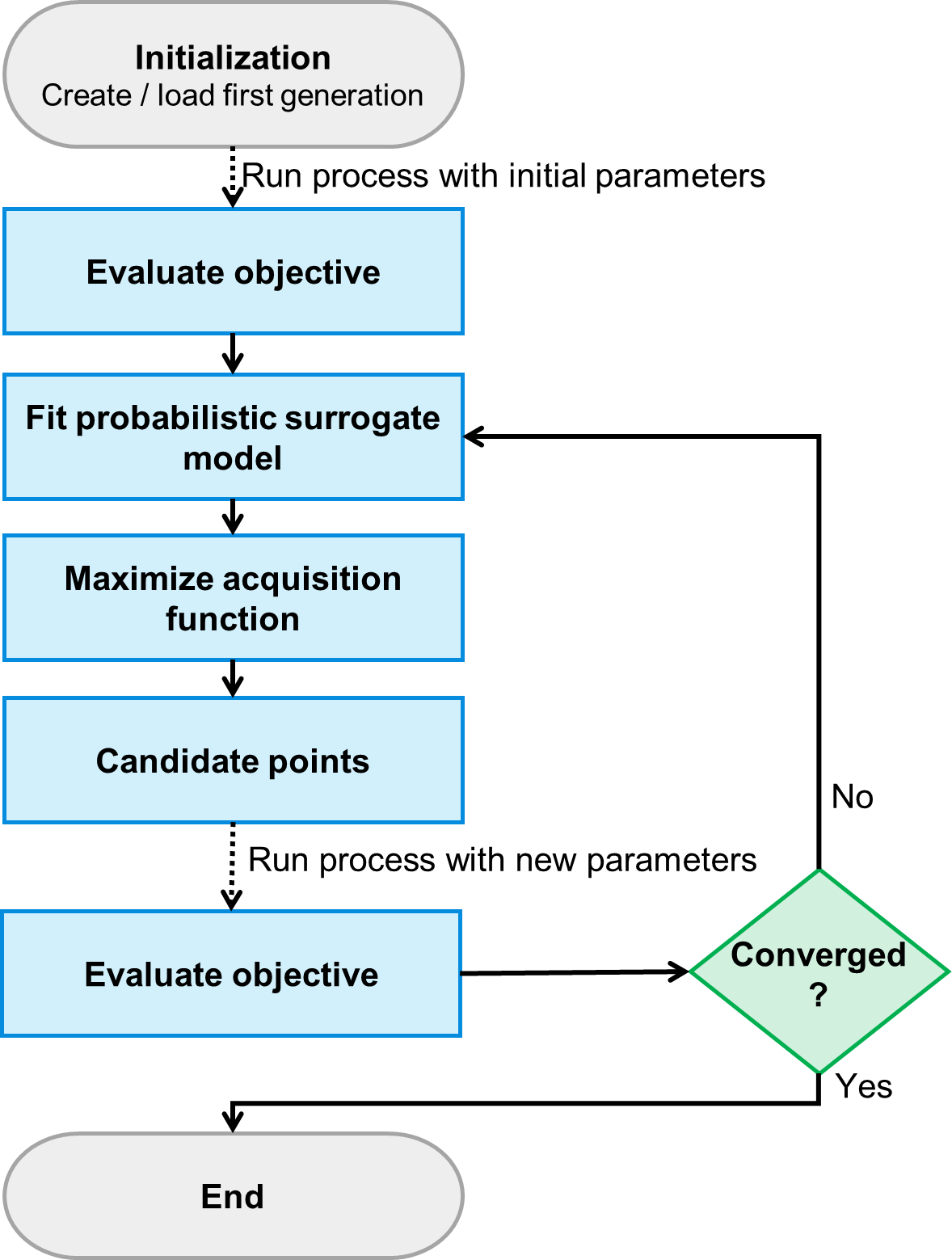


**Figure S5:** Flow-chart of the utilized Bayesian Optimization (BO) algorithm.

**Table S3**: Tested hyperparameters of the Bayesian Optimization (BO) algorithm in the in-silico algorithm screening.

| **Hyperparameter** | **Tested values** |
| --- | --- |
| Acquisition Function | Expected Improvement (EI)  Probability of Improvement (PI)  Upper Confidence Bound (UCB)  Epsilon Greedy (EG) |
| Kernel Function | Radial Basis Function (RBF)  Rational Quadratic (RQ)  Matérn 1/2  Matérn 3/2  Matérn 5/2 |

**2.4 Simulated Annealing (SA)**

Simulated Annealing (SA) is a probabilistic optimization technique inspired by the annealing process in metallurgy, where a material is heated and then gradually cooled to reduce defects and improve its structural integrity. The goal of the algorithm is to find the global optimum of an objective function by exploring the solution space and gradually refining the search towards promising regions.

**1. Initialization**

- The algorithm begins by initializing a set of candidate solutions (positions) within the defined parameter bounds, including pH, salt concentration, cosubstrate concentration, organic solvent concentration, and temperature. Each position represents a set of enzyme reaction conditions. For the in-silico algorithm testing, the initial generations are loaded from a predefined set of parameter combinations.
- An initial temperature T_0_ is set, which controls the acceptance probability of worse solutions during the optimization process.

**2. Evaluate Objective Function**

- The objective function involves linear interpolation of the specific activity based on a pre-existing dataset of enzyme reaction results. These initial evaluations provide baseline scores for each position.

**3. Add Random Perturbation to Current Positions**

- A uniform random perturbation is applied to each parameter i of the current positions $x_{i}^{t}$to generate new candidate solutions $x_{i}^{t+1}$. The perturbation is scaled by a predefined step size $S$, which determines the magnitude of the change (Eq. S15).

$x_{i}^{t+1}= x_{i}^{t}+r_{i}S$ (S15)

- $r_{i}$ is a random number drawn from a uniform distribution in [0,1].
- $S$is a percentage of the parameter range

**4. Evaluate Objective Function for New Positions**

- The objective function is evaluated for the newly generated positions, resulting in a new set of scores. These scores are compared with the previous scores to determine whether the new positions should be accepted.

**5. Calculate Difference and Accept New Position**

- The difference between the new score and the old score is calculated (Eq. S16).

$\Delta=f\left( x^{t+1} \right)-f(x^{t})$ (S16)

- The decision to accept the new position is made based on the following criteria:
  - If the new score is better (Δ > 0), the new position is always accepted.
  - If the new score is worse (Δ ≤ 0), the new position may still be accepted with a probability proportional to $exp\left( \frac{\Delta}{T} \right)$. This probabilistic acceptance allows the algorithm to escape local optima by occasionally accepting worse solutions, especially at higher temperatures.

**6. Cool Down the Temperature**

- After each iteration, the temperature is reduced according to a cooling rate $c_{T}$ in (0,1) (Eq. S17). The cooling rate controls how quickly the temperature decreases over time. A slower cooling rate allows for more thorough exploration of the solution space, while a faster cooling rate accelerates convergence.

$T^{t+1}=T^{t}c_{T}$ (S17)

**7. Convergence Check**

- The algorithm checks for convergence by monitoring the improvement in the best score over a fixed number of iterations. Convergence is achieved if there is no significant improvement in the best score over several consecutive iterations, or if a maximum number of iterations is reached. Once convergence is detected, the algorithm terminates and returns the best solution found.

**Evaluated Hyperparameters**

For screening different SA algorithm variants, 3 hyperparameters were varied. These include the initial temperature $T_{0}$, the cooling rate $c_{T}$ and the step size S (Table S4).


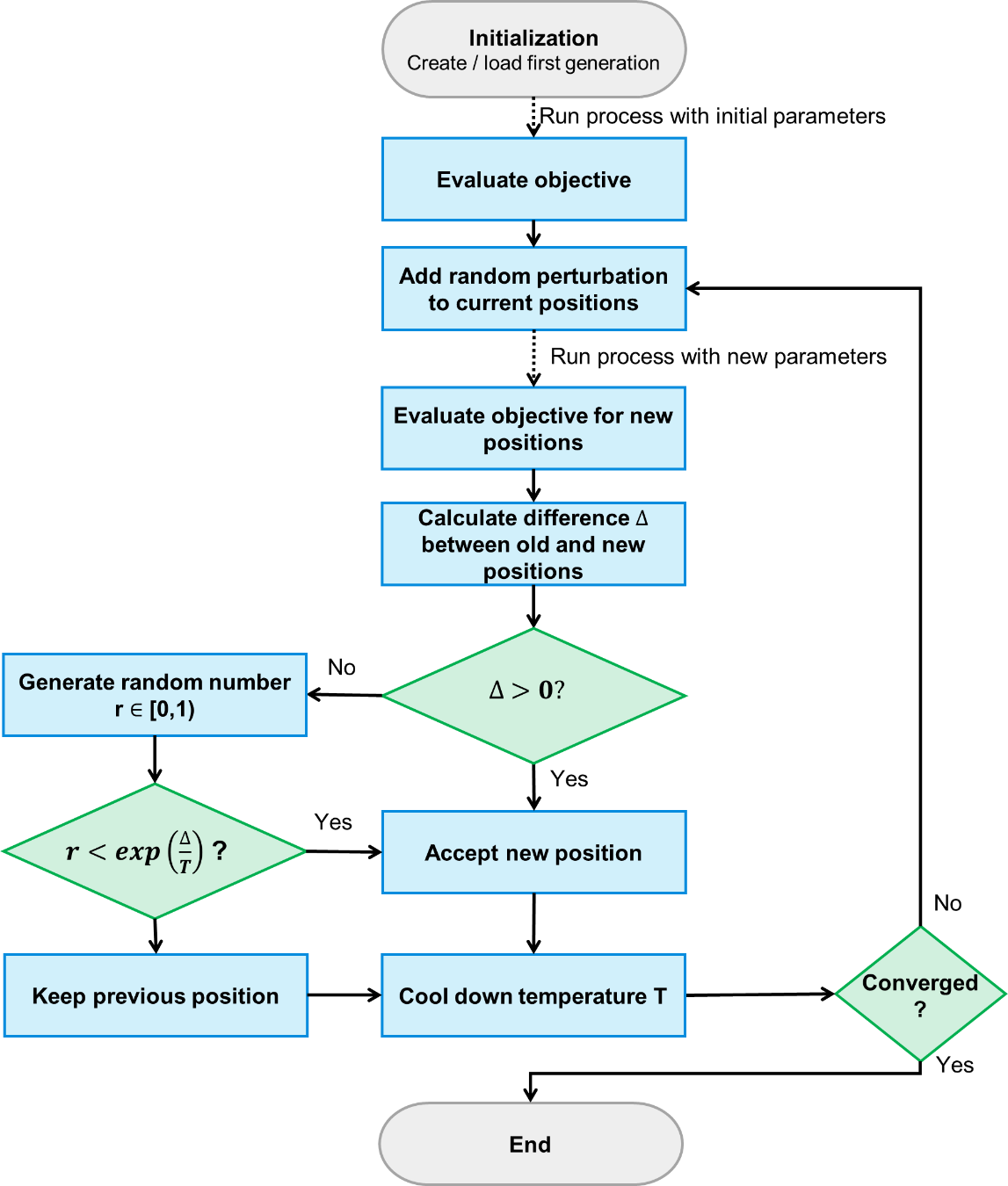


**Figure S6:** Flow-chart of the utilized Simulated Annealing (SA) algorithm.

**Table S4:** Tested hyperparameters of the Simulated Annealing (SA) algorithm in the in-silico algorithm screening.

| **Hyperparameter** | **Tested values** |
| --- | --- |
| $T_{0}$ | 45000, 90000, 225000, 450000, 1800000 |
| $c_{T}$ | 0.7, 0.8, 0.9, 0.95, 0.99 |
| $S_{0}$ | 0.05, 0.1, 0.2, 0.4, 0.6, 0.8 |

**Random Search (RS)**

Random search is a simple optimization algorithm that generates random candidate solutions within predefined bounds and evaluates their performance using an objective function. Unlike more sophisticated methods that explore the solution space in a guided manner, random search relies purely on stochastic sampling, which makes it robust in high-dimensional spaces and less prone to getting stuck in local optima.

**1. Initialization**

- The algorithm begins by initializing a set of candidate solutions (positions) within the defined parameter bounds, including pH, salt concentration, cosubstrate concentration, organic solvent concentration, and temperature. Each position represents a set of enzyme reaction conditions. For the in-silico algorithm testing, the initial generations are loaded from a predefined set of parameter combinations.

**2. Evaluate Objective Function**

- The objective function involves interpolation of the specific activity based on a pre-existing dataset of enzyme reaction results. These initial evaluations provide baseline scores for each position.

**3. Generate Random New Solutions**

- In each iteration, a new set of random solutions is generated. These solutions are created by randomly sampling each parameter within its respective bounds and rounding to the required precision.
- To avoid evaluating duplicate solutions, a set is maintained to track previously tested combinations. If a generated solution already exists in the set, a new solution is sampled until a unique one is found.

**4. Evaluate Objective Function for New Solutions**

- The newly generated solutions are evaluated using the objective function, and their scores are computed.

**6. Convergence Check**

- The algorithm runs for a fixed number of iterations (e.g., 50 iterations). Convergence is implicitly achieved when no significant improvement is observed over several iterations, or when the maximum number of iterations is reached. After completing the specified iterations, the best solution found during the entire process is returned.

**
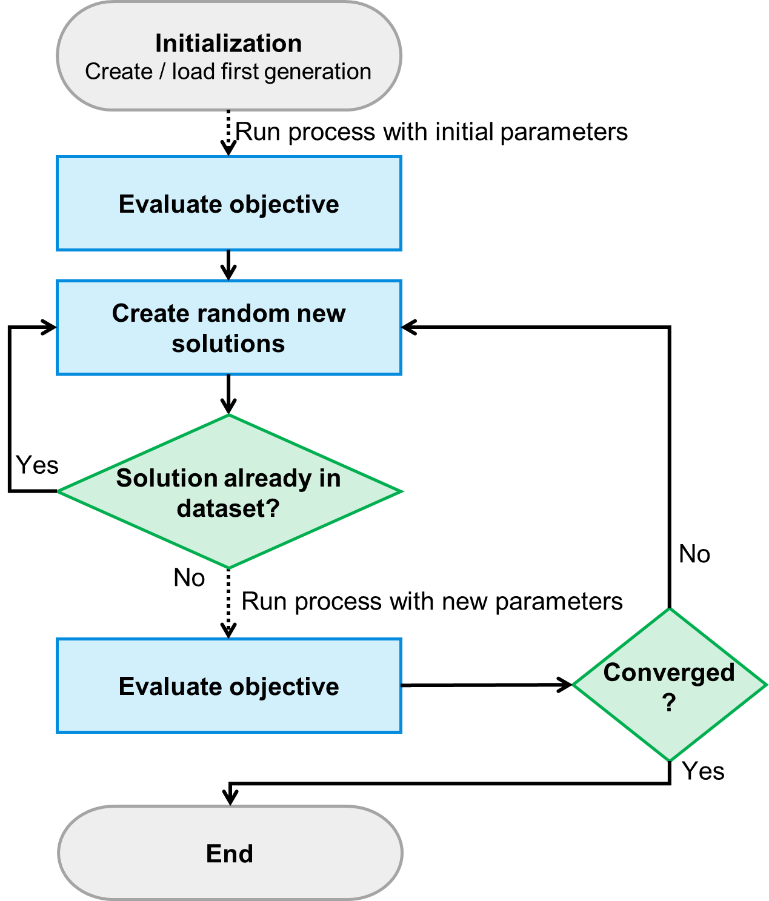
**

**Figure S7:** Flow-chart of the utilized random search (RS) algorithm.

**Response Surface Modelling (RSM) using Design of Experiments (DoE)**

Response Surface Methodology (RSM) is an optimization technique that leverages **Design of Experiments (DoE)** to systematically model and explore the relationships between input variables and a response variable. It is particularly effective when the objective is to find the optimal conditions for a process with multiple interacting parameters. RSM involves generating a structured experimental design—often using a **Central Composite Design (CCD)** or other factorial designs—to ensure comprehensive coverage of the parameter space. By conducting a sequence of well-planned experiments, fitting a mathematical model (usually a quadratic polynomial), and iteratively refining the search space around the predicted optimum, RSM efficiently identifies optimal or near-optimal conditions. The iterative process helps in narrowing the parameter bounds and improving prediction accuracy with each successive iteration.

1. Start and Create Central Composite Design (CCD)

- The algorithm begins with an initial iteration i=0 by generating a Central Composite Design (CCD) within a defined parameter space. The CCD includes center points, factorial points, and star points, ensuring good coverage of the parameter space. The parameter space is defined by lower and upper bounds for each of the five variables: pH, salt concentration, cosubstrate concentration, organic solvent concentration, and temperature.

2. Run Process and Evaluate Objective

- Each parameter set generated by the CCD is evaluated using the objective function, which predicts the mean specific reaction rate through interpolation based on experimental data. The objective function assigns a response value to each parameter set, representing the process outcome under those conditions.

3. Fit Quadratic Model

- Once all responses are evaluated, a quadratic model is fitted to the data using Ordinary Least Squares (OLS) regression. This model includes linear, interaction, and quadratic terms to capture the relationships between the input variables and the response. The general form of the quadratic model is with n parameters $x_{i, i=1\ldots n}$ is given by Eq. S18.

$f\left( x \right)=\beta_{0}+\sum_{i=1}^{n} \beta_{i}x_{i}+\sum_{i=1}^{n} \beta_{ii}{x_{i}}^{2}+\sum_{i=1}^{n-1} {\sum_{j=i+1}^{n} \beta_{ij}x}_{i}x_{j}$ (S18)

4. Calculate Optimum

- Using the fitted quadratic model, optimization is performed using the L-BFGS-B algorithm, a limited-memory quasi-Newton method tailored for optimization problems with simple box constraints^[[1]](#footnote-1)^, to find the set of parameters that maximizes the predicted response.

5. Update Bounds and Create New CCD

- Once the optimum is calculated, a new CCD is generated around the predicted optimum. The bounds of the parameter space are updated to focus the search on a smaller region centered around the optimum. This refinement reduces the search range in subsequent iterations, improving the accuracy of the optimization.
- The parameter ranges are halved and the bounds are updated using the following logic:
- If the optimum is near the current bounds, the new bounds are shifted outward to include more of the parameter space.
- If the optimum is well within the current bounds, the new bounds are narrowed symmetrically around the optimum.

6. Repeat Process for N iterations

- The above steps are repeated for a fixed number of iterations N. In each iteration, the parameter space becomes progressively smaller. The iterations continue until the maximum number of iterations is reached or convergence criteria are met. In this study 3 iterations for the RSM were used to compare it to the other algorithms.


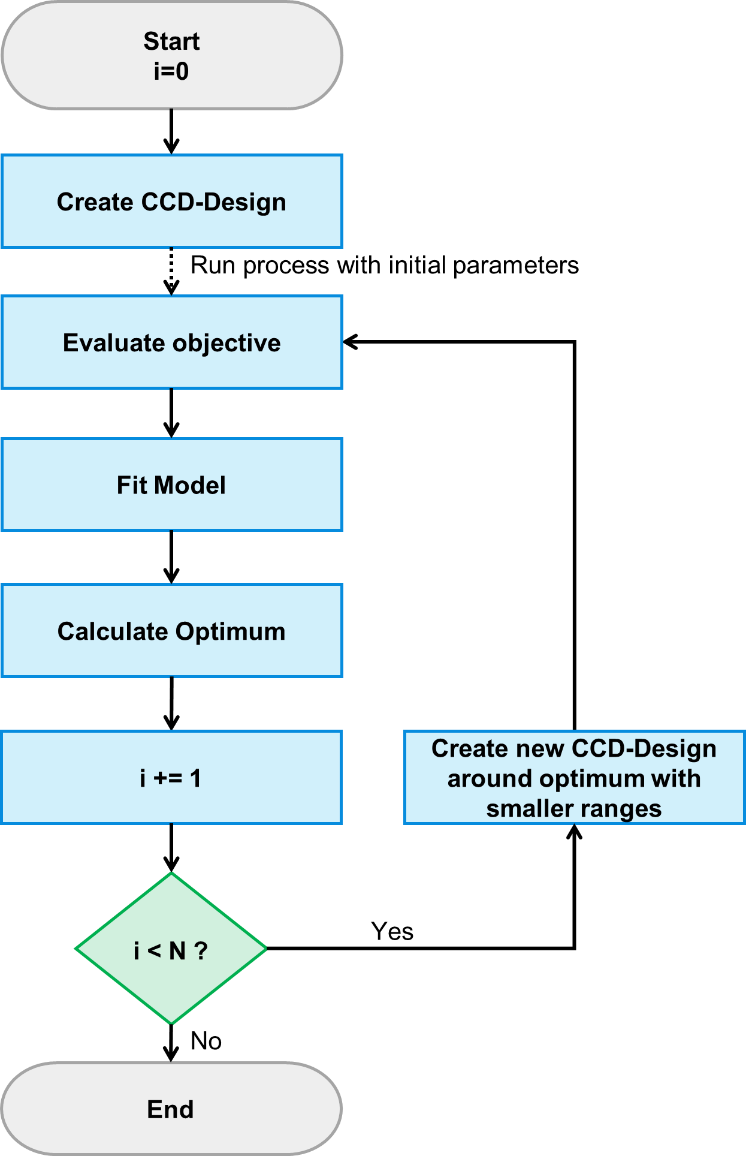


**Figure S8:** Flow-chart of the Response Surface Modelling (RSM).

1. **Autonomous enzymatic reaction optimization – Additional material**
   1. **Posterior variance analysis**

**
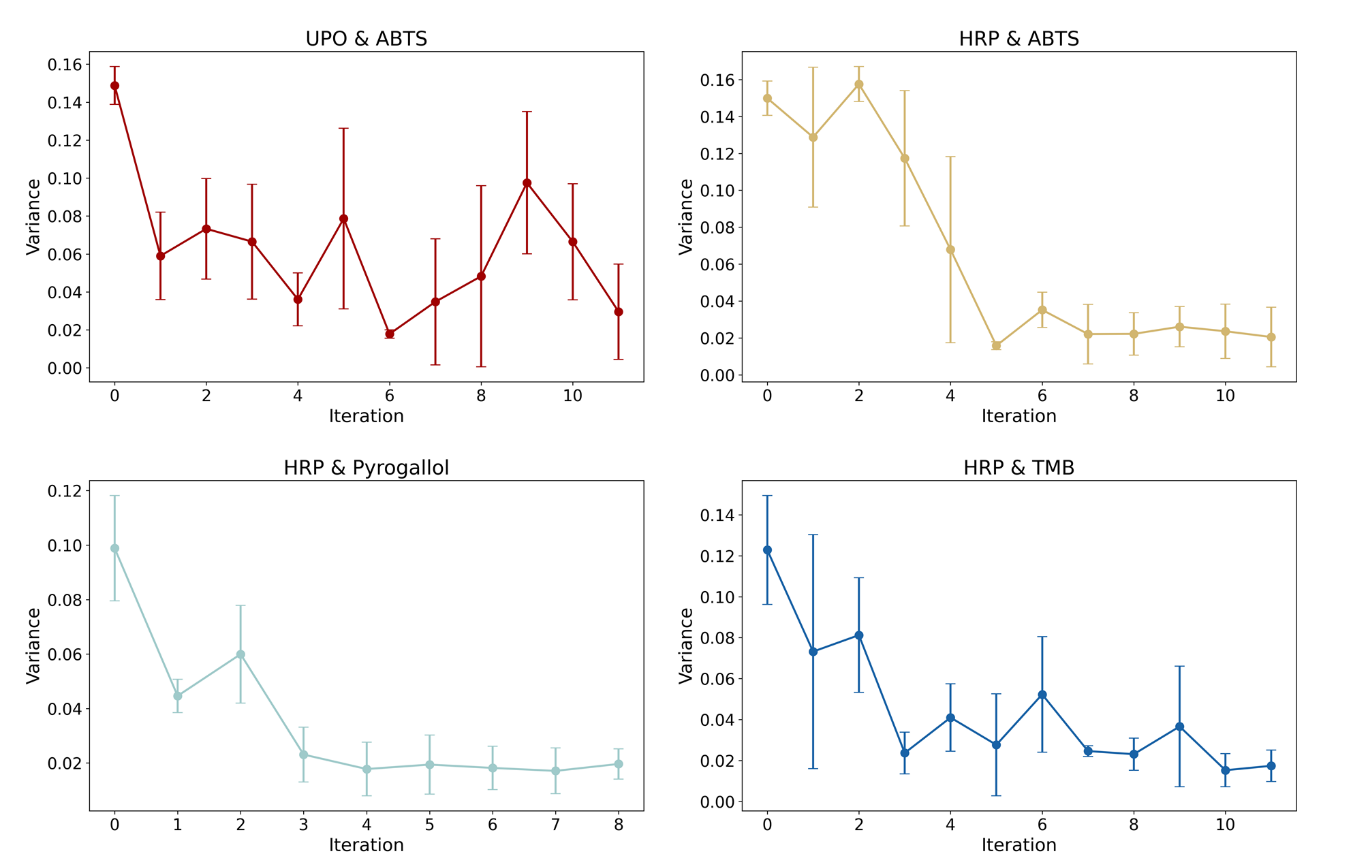
**

**Figure S9:** Posterior variance in the autonomous enzymatic reaction condition optimization experiments on the SDL for the enzyme-substrate-pairings (a) UPO-ABTS, (b) HRP-ABTS, (c) HRP-Pyrogallol and (d) HRP-TMB. The shown values represent the mean and standard deviation of the 8 tested parameter combinations in each iteration.

In Gaussian Process (GP) regression, the **posterior variance** quantifies the model's uncertainty about its predictions. It reflects how confident the GP model is in its predictions at each input point. The decreasing posterior variance in all optimization campaigns indicates increasing certainty of the model. The minimal achievable posterior variance is the variance arising from the experiment itself. Assuming an experimental relative standard deviation σ of the enzyme assays between 0.1 and 0.15, a posterior variance σ^2^ of minimally 0.01 to 0.0225 can be achieved. This value was approached in all optimization runs, especially for the experiments using HRP, where the posterior variance reached a plateau at approximately 0.02, indicating convergence of the BO.

**
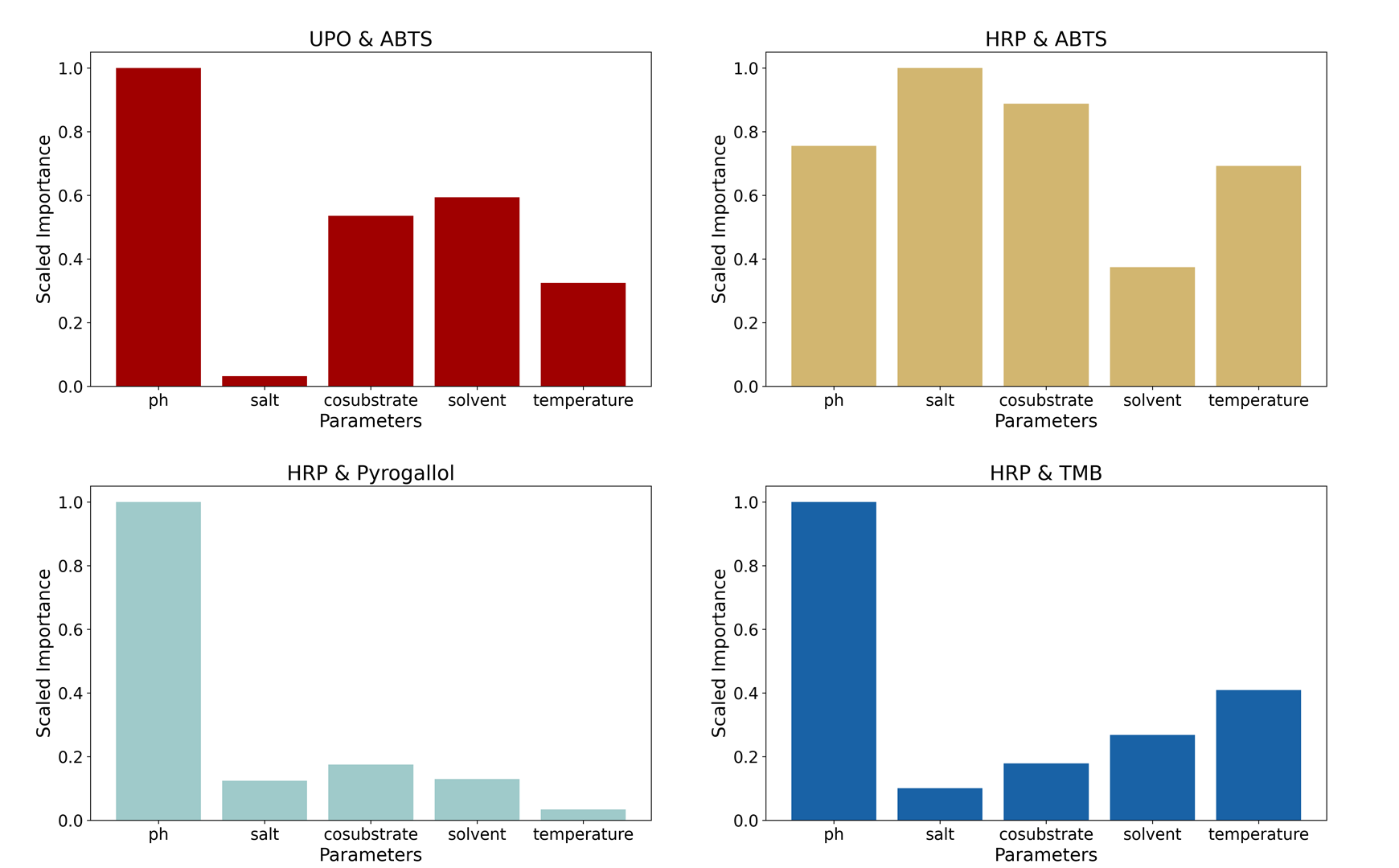
**

**Figure S10:** Parameter importances determined by Random Forest Regression in the autonomous enzymatic reaction condition optimization experiments on the SDL for the enzyme-substrate-pairings (a) UPO-ABTS, (b) HRP-ABTS, (c) HRP-Pyrogallol and (d) HRP-TMB.

1. **Linear interpolation surrogate model confidence and uncertainty quantification**

The reliability and uncertainty of the surrogate model used for algorithm benchmarking in this study are closely linked to the sampling density and noise characteristics of the experimental dataset. Here, we present a critical assessment of the linear interpolation approach, quantify its uncertainty, and discuss its implications for benchmarking optimization algorithms.

The surrogate model was constructed using linear interpolation of measured enzymatic activities in a 5-dimensional design space. In the following, the uncertainty assessment is shown for varying pH and temperature, while other parameters were fixed at their optimal values (c_H₂O₂_ = 8.75 mM, c_Na₂SO₄_ = 120 mM, c_ACN_ = 0% v/v). In regions densely covered by experimental data, the interpolation is well-constrained, resulting in an accurate representation of the true response surface. This is visualized in the interpolated mean activity surface (**Figure S11(a)),** where the global optimum and response gradients are clearly resolved.

Conversely, in sparsely sampled regions—such as the extremes of pH and temperature—confidence in the interpolated values is reduced. These areas may exhibit artificial linearity or smoothing, which does not necessarily reflect true enzymatic behavior. The standard deviation surface (Figure **S11(b)**) reveals that measurement noise is highest in regions of maximal enzymatic activity, while the coefficient of variation (CV, Figure **S11(c)**) highlights regions where the signal-to-noise ratio is lowest, typically near the boundaries of the design space.

To explicitly visualize a proxy for interpolation uncertainty, we mapped the Euclidean distance from each interpolated grid point to its nearest experimental neighbor (Figure **S11(d)**). High values in this distance map indicate under-sampled regions, which are expected to have higher interpolation uncertainty. To provide another simple proxy for overall uncertainty, we multiplied the interpolated standard deviation by the nearest neighbor distance at each point (SD × distance, Figure **S11(e)**). This metric highlights regions where both experimental noise and sparse sampling combine to reduce the reliability of interpolated predictions, particularly at low pH (2.5) and high temperature (50 °C).

To assess the robustness of the optimization landscape in the presence of experimental noise, we generated five random realizations of the interpolated mean surface by adding random values within ±1 SD to each grid point (**Figure S12**). All realizations preserved the main optimum and overall landscape features. Thus, while local uncertainties are present, the global landscape—including the optimum location—remains robust against the level of noise encountered in our dataset. This still holds true, when the random noise is doubled to ±2 SD (**Figure S13**)

We acknowledge two main limitations of this approach. First, in regions far from the optimum—such as at the edges of the design space or at extremes of pH and temperature—sampling is sparse, and interpolation may produce artificially smooth or linear behavior that does not accurately capture expected biochemical profiles. Second, linear interpolation does not provide formal confidence intervals; our SD × distance metric is only a heuristic.

Despite these limitations, the surrogate model is appropriate for its intended use: benchmarking optimization algorithms under realistic, noisy conditions. The focus of this study is on comparing algorithmic performance rather than generating a predictive process model. The robustness analyses show that key features of the optimization landscape are preserved, and the location of the global optimum is consistent with both literature values and experimental validation.


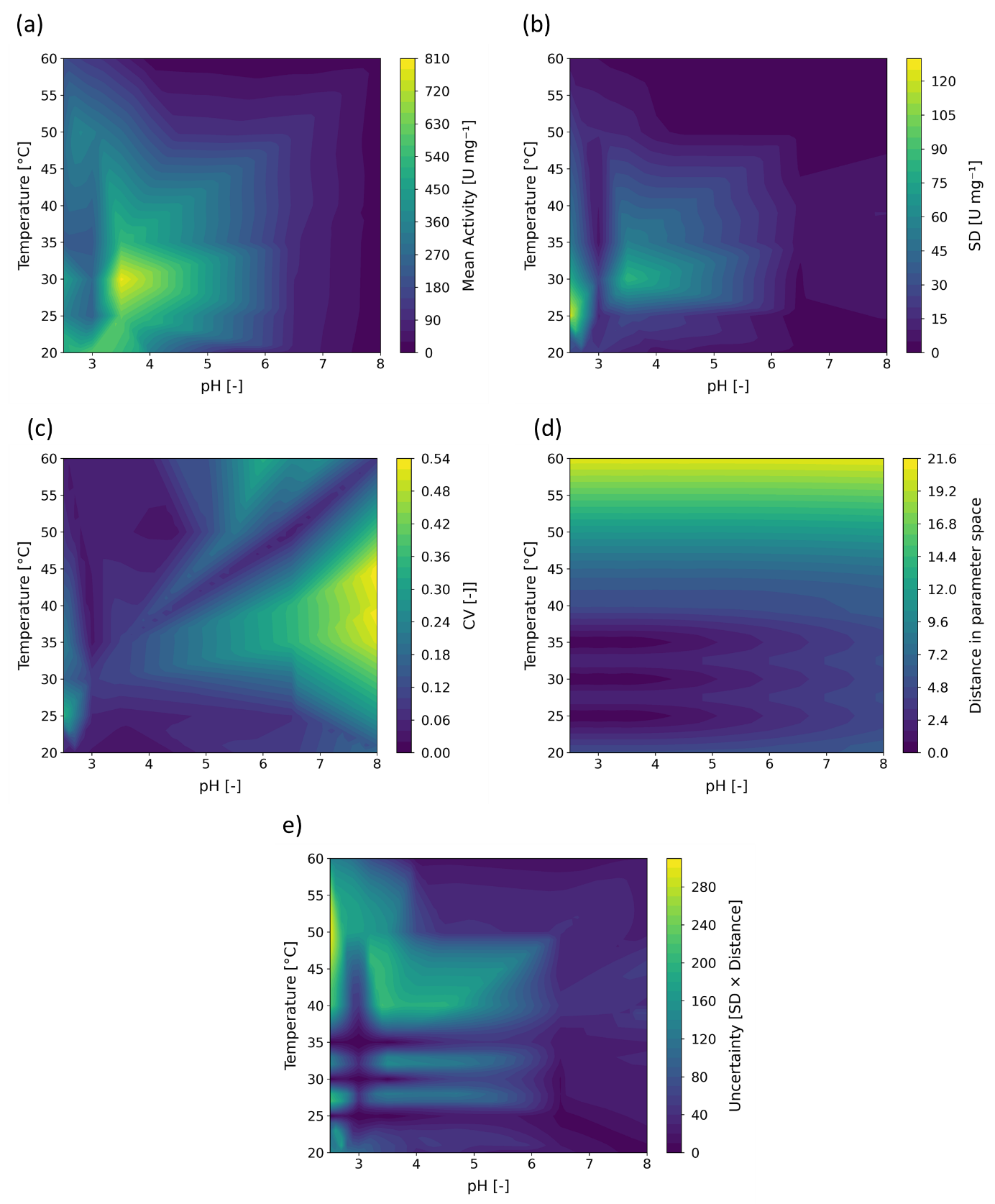


**Figure S11: Visualization of the linear interpolation surrogate model and uncertainty proxies for the enzymatic activity landscape as a function of pH and temperature (other parameters fixed at optimal values:** c_H₂O₂_ = 8.75 mM, c_Na₂SO₄_ = 120 mM, c_ACN_ = 0% v/v**).** (a) **Interpolated mean activity:** Response surface showing the interpolated mean enzymatic activity. (b) **Interpolated standard deviation (SD):** Surface showing experimental variability as interpolated SD. (c) **Interpolated coefficient of variation (CV):** Relative measurement uncertainty, highlighting increased noise at the design space boundaries. (d) **Nearest neighbor distance:** Euclidean distance to the closest experimental data point in parameter space, serving as a proxy for interpolation confidence. (e) **Interpolation uncertainty proxy (SD × distance):** Product of SD and nearest-neighbor distance, used as a visual metric for regions of high interpolation uncertainty.


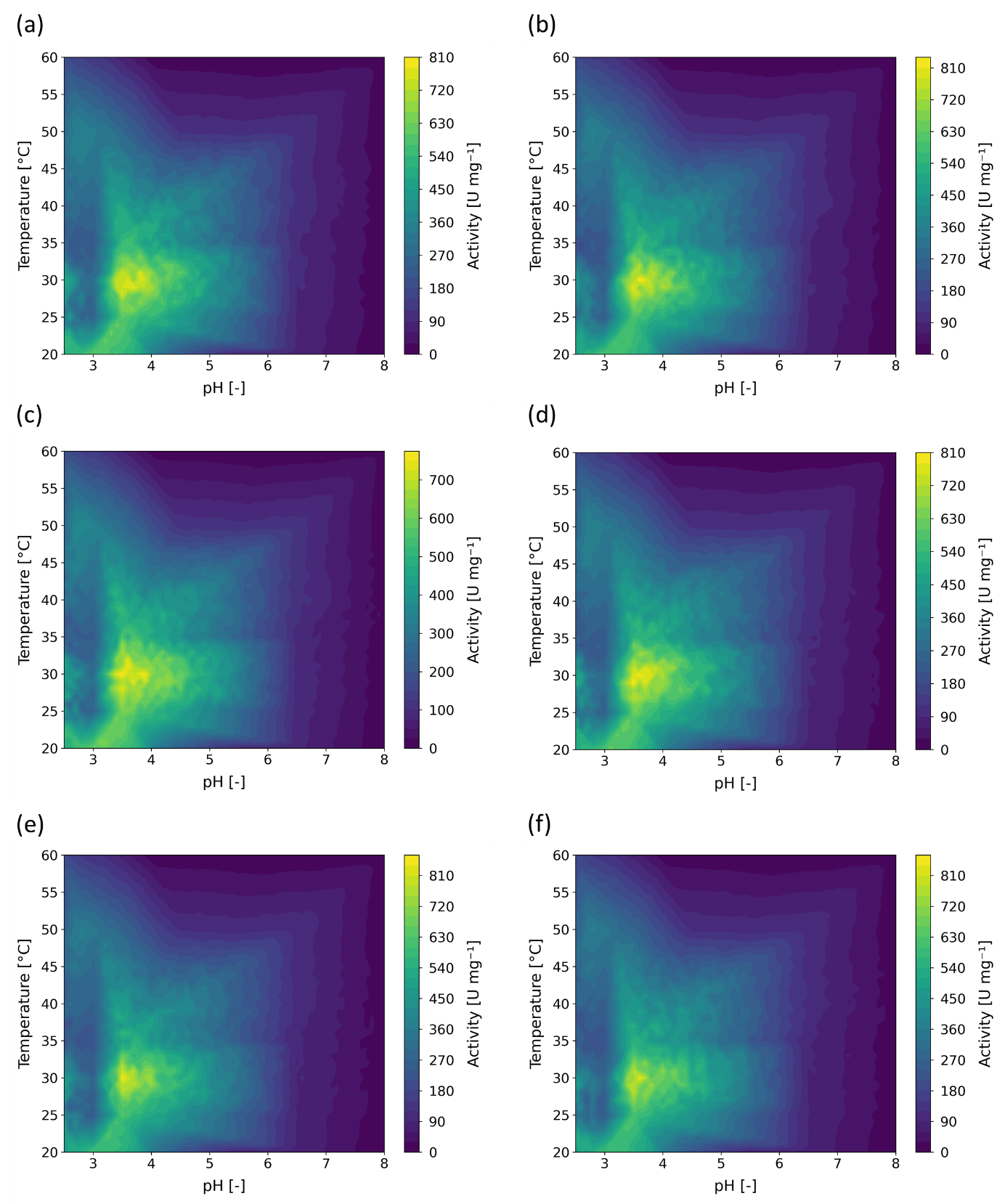


**Figure S12:** Robustness analysis of the interpolated landscape to experimental noise (±1 SD). Six independent realizations (a)-(f) of the interpolated mean activity surface **as a function of pH and temperature (other parameters fixed at optimal values:** c_H₂O₂_ = 8.75 mM, c_Na₂SO₄_ = 120 mM, c_ACN_ = 0% v/v**)** were generated by adding random noise within ±1 standard deviation at each grid point. All surfaces consistently recover the main optimum and preserve the global landscape features, indicating that the optimization landscape is robust against the typical experimental variability observed in this study.

**
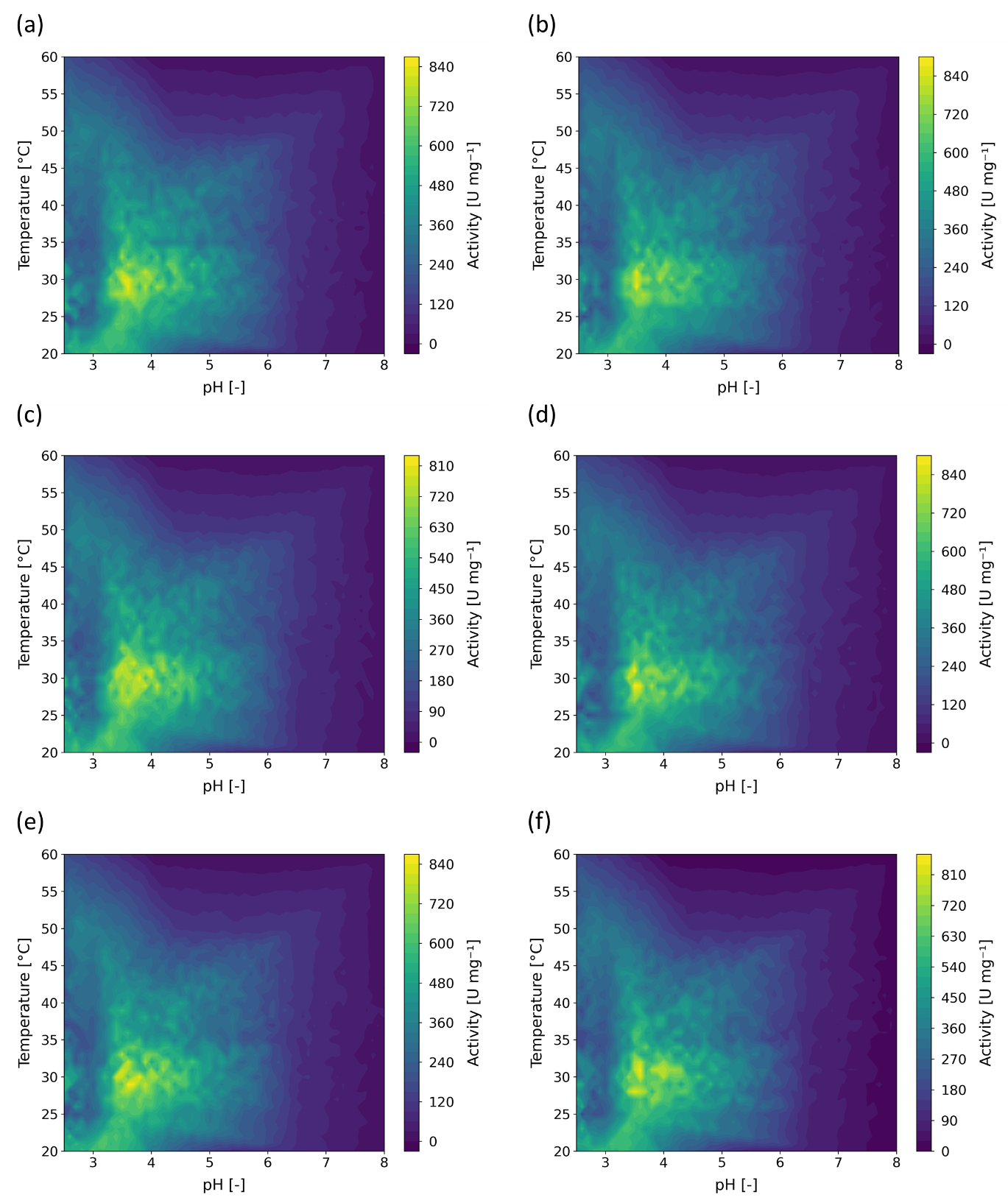
**

**Figure S13:** Robustness analysis of the interpolated landscape to increased experimental noise (±2 SD). Six independent realizations (a)-(f) of the interpolated mean activity surface **as a function of pH and temperature (other parameters fixed at optimal values:** c_H₂O₂_ = 8.75 mM, c_Na₂SO₄_ = 120 mM, c_ACN_ = 0% v/v**)** were generated by adding random noise within ±2 standard deviation at each grid point. Even with this amplified noise, the global optimum and the overall shape of the landscape remain preserved, confirming the robustness of the landscape to substantial local uncertainty.

1. **Zhu, C., Byrd, R. H., Lu, P., & Nocedal, J. (1997).** Algorithm 778: L-BFGS-B: Fortran subroutines for large-scale bound-constrained optimization. ACM Transactions on Mathematical Software (TOMS), 23(4), 550–560. https://doi.org/10.1145/279232.279236 [↑](#footnote-ref-1)
